# A cap 0-dependent mRNA capture method to analyze the yeast transcriptome

**DOI:** 10.1093/nar/gkac903

**Published:** 2022-10-19

**Authors:** Martyna Nowacka, Przemysław Latoch, Matylda A Izert, Natalia K Karolak, Rafal Tomecki, Michał Koper, Agnieszka Tudek, Agata L Starosta, Maria W Górna

**Affiliations:** Biological and Chemical Research Centre, Department of Chemistry, University of Warsaw, Warsaw, Warsaw 02-093, Poland; Institute of Biochemistry and Biophysics, Polish Academy of Sciences, Warsaw, Warsaw 02-106, Poland; Polish-Japanese Academy of Information Technology, Warsaw, Warsaw 02-008, Poland; Biological and Chemical Research Centre, Department of Chemistry, University of Warsaw, Warsaw, Warsaw 02-093, Poland; Biological and Chemical Research Centre, Department of Chemistry, University of Warsaw, Warsaw, Warsaw 02-093, Poland; Nencki Institute of Experimental Biology, Polish Academy of Sciences, Warsaw, Warsaw 02-093, Poland; Institute of Biochemistry and Biophysics, Polish Academy of Sciences, Warsaw, Warsaw 02-106, Poland; Institute of Genetics and Biotechnology, Faculty of Biology, University of Warsaw, Warsaw, Warsaw 02-106, Poland; Institute of Genetics and Biotechnology, Faculty of Biology, University of Warsaw, Warsaw, Warsaw 02-106, Poland; Institute of Biochemistry and Biophysics, Polish Academy of Sciences, Warsaw, Warsaw 02-106, Poland; Institute of Biochemistry and Biophysics, Polish Academy of Sciences, Warsaw, Warsaw 02-106, Poland; Biological and Chemical Research Centre, Department of Chemistry, University of Warsaw, Warsaw, Warsaw 02-093, Poland

## Abstract

Analysis of the protein coding transcriptome by the RNA sequencing requires either enrichment of the desired fraction of coding transcripts or depletion of the abundant non-coding fraction consisting mainly of rRNA. We propose an alternative mRNA enrichment strategy based on the RNA-binding properties of the human IFIT1, an antiviral protein recognizing cap 0 RNA. Here, we compare for *Saccharomyces cerevisiae* an IFIT1-based mRNA pull-down with yeast targeted rRNA depletion by the RiboMinus method. IFIT1-based RNA capture depletes rRNA more effectively, producing high quality RNA-seq data with an excellent coverage of the protein coding transcriptome, while depleting cap-less transcripts such as mitochondrial or some non-coding RNAs. We propose IFIT1 as a cost effective and versatile tool to prepare mRNA libraries for a variety of organisms with cap 0 mRNA ends, including diverse plants, fungi and eukaryotic microbes.

## Graphical Abstract

**Graphical Abstract:**
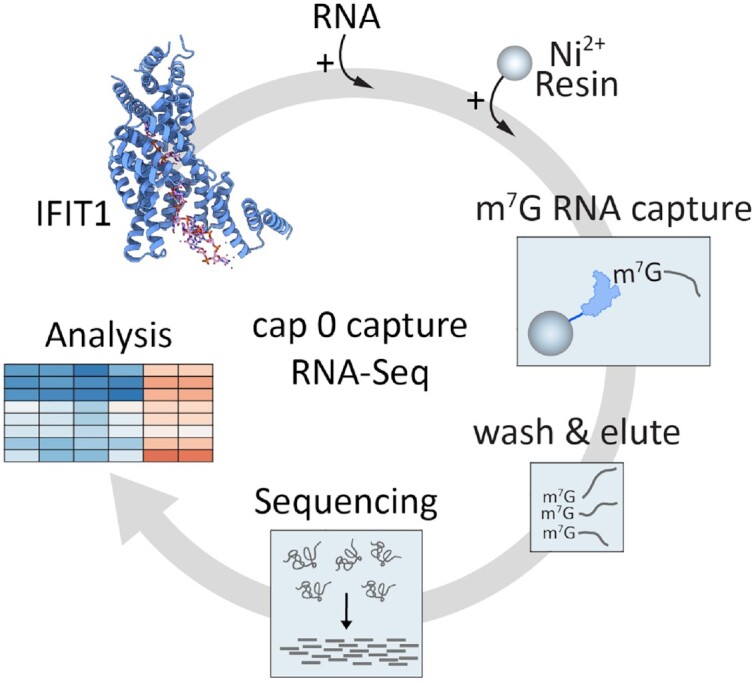
A new method for mRNA enrichment based on the capture of Cap 0 groups on IFIT1 protein immobilized on a resin.

## INTRODUCTION

RNA sequencing (RNA-seq) is an approach to profile transcriptomes with deep sequencing technologies. RNA-seq allows detection and analysis of a variety of RNA species within a sample, including mRNA, long and small non-coding RNA, as well as pathogen RNA. Transcriptomic analysis gives insight into many cellular processes and provides information about gene expression level, gene fusions, alternative splice variants, mutations, transcript isoforms in terms of their 5′ and 3′ ends, and many other features. In order to perform RNA-seq-based analysis of the protein coding transcriptome, mRNAs need to be efficiently separated from other RNA species, especially from the highly abundant ribosomal RNA (rRNA) which account for the majority (as much as 80–90%) of total RNA in the cell (1). This is typically accomplished by selective hybridization or priming-based methods such as rRNA depletion (ribodepletion) or oligo-dT (which hybridizes to polyA tails) mRNA capture, all of which may have their own strengths and biases, including limitations of the range of species for which a commercial set of probes may be available (2–5).

**Figure 1.**
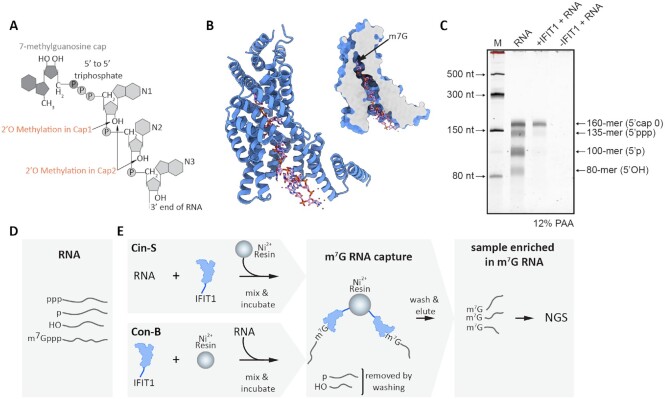
IFIT1 protein properties and the pull-down procedure. (**A**) The cap 0 structure characteristic for yeast mRNA, encompassing the m^7^G moiety at the 5′ end connected by a triphosphate bridge to the body of the mRNA. In cap 0, the 2′ hydroxyl groups of the first and second nucleotides remain unmodified, whereas the higher-methylated caps of vertebrates contain additional methyl groups in the 2′O position: cap 1 is methylated in the first nucleotide, and cap 2 in both the first and the second nucleotide of mRNA. (**B**) Crystal structure of the IFIT1 monomer bound to cap 0-RNA (based on PDB ID: 6C6K (18)) and location of the narrow pocket of IFIT1 which binds the 5′ cap (m^7^G) group. (**C**) Pull-down on a Nickel resin with immobilized IFIT1 selects cap 0-modified RNA. Denaturing PAGE analysis of: four *in vitro* transcribed RNA molecules (a mix of 80-mer, 100-mer, 135-mer and 160-mer) with different 5′-end modifications (‘RNA’), RNA molecules recovered from the complexes with IFIT1 after pull-down (‘+IFIT1 + RNA’), and after the pull-down procedure on a resin without IFIT1 as a negative control (‘-IFIT1 + RNA’). M – RNA size marker. (**D**) A pool of RNA may differ by various 5′ end modifications, such as hydroxyl (OH), triphosphate (ppp), monophosphate (p) or cap 0 (m^7^G) moieties. (**E**) Pull-down of RNA using IFIT1 was performed in two alternative approaches, differing in the order of the first two steps. Cin-S (RNA-IFIT1 ‘complex in solution’) approach starts by incubation of free IFIT1 with RNA, followed by immobilization of the complexes on the Nickel resin. In Con-B (RNA-IFIT1 ‘complex on beads’), IFIT1 is first immobilized on the Nickel resin before the addition of RNA. An incubation step allows the formation of specific IFIT1-RNA complexes and their capture on the beads. RNA not recognized by IFIT1 such as those with 5′ OH, p and ppp groups, are removed in the washing step, leading to selective enrichment of the bound cap 0 (m^7^G) RNA. Next, cap 0 RNA isolated from IFIT1-coated beads is ready for downstream assays or next generation sequencing (NGS).

With the widespread use of RNA-seq and its various applications, there is a need for developing new, alternative mRNA enrichment methods. Proteins with unique RNA binding properties seem to be a viable and robust solution for mRNA sample enrichment prior to RNA-seq (6). The cap-dependent RNA purification systems have already been introduced based on the eukaryotic initiation factor 4E (eIF4E) which binds the 5′ N7-methylguanosine GTP moiety of the m^7^G(5′)ppp(5′)N cap of eukaryotic mRNA (Figure 1A), as well as on the eIF4E(K119A) mutant version which has a higher affinity than wild-type protein and is capable of binding also trimethylated cap structures (m_3_^2,2,7^GTP) (7,8). For instance, global mRNA captured using eIF4E was successfully sequenced and the obtained data was used for *de novo* transcriptome assembly in *Xenopus laevis* (9). Conveniently, using a cap-dependent mRNA capture method preferentially enables studies focused on the variable 3′ end polyadenylation status (10) or TOP mRNA (11). Recently, the murine eIF4E in fusion with B4E protein and lactamase and in conjunction with poly-deoxythymidine oligonucleotide was used in a biosensor designed to simultaneously detect both, the polyadenylation status and the presence of cap structures on mRNAs such as mRNA vaccines (12). eIF4E is the only protein used so far for cap-dependent mRNA enrichment or detection, yet is sensitive to cap methylation (requires 5′ N7-methylguanosine) and may not work with all cap analogs or synthetic GpppRNA.

Characterized over a decade later than eIF4E(K119A) (7), Interferon-Induced protein with Tetratricopeptide repeats 1 (IFIT1) is a cap-dependent RNA-binding protein (Figure 1B) produced in cells in response to a viral infection as part of the vertebrate innate immune response (13–16). The IFIT family includes five human paralogues (IFIT1, 1B, 2, 3, 5), each containing only one domain (∼55 kDa) comprising helical tetratricopeptide repeats (TPR) (17–19). The antiviral role of human IFITs depends on the ability to discriminate the ‘non-self’ features of pathogen RNA in the cell, and in this instance, IFIT1 has been demonstrated to recognize cap 0-containing RNAs (i.e. m^7^G-capped RNA without further 2′O-methylation of the first and second nucleotide; Figure 1A) and to some extent also RNA with a triphosphate moiety (pppRNA) or even cap 1 (m^7^GpppNm-RNA) (14,15,17,20,21). In addition, IFIT1 requires a few unpaired nucleotides at the 5′-end due to the narrow dimensions of the RNA-binding pocket (Figure 1B), which has a form of a positively-charged tunnel with a separate hydrophobic extension for cap binding (17). Importantly, IFIT1 does not have sequence requirements nor depends on N7 methylation, so that in general it binds both GpppRNA and m^7^GpppRNA in a largely sequence-independent manner (17,20). Several crystal structures show that IFIT1 undergoes only a modest conformational change upon RNA binding and the sequestered RNA molecule is then prevented from translation by displacement of cap-dependent translation initiation factors such as eIF4E, suggesting very efficient capped RNA binding and competition with other cap-binding factors *in vivo* (16–19).

Here, we demonstrate that human IFIT1 can be successfully applied for mRNA capture in *Saccharomyces cerevisiae* prior to RNA-seq library preparation. Baker's yeast, as a model lower eukaryote, typically has RNAs transcribed by RNA polymerase II modified co-transcriptionally with cap 0 structure, since capping is obligatory for stability, export and processing of mRNA as well as for protein synthesis. Comparison of our method with a commercial RiboMinus rRNA depletion kit (RM) shows that IFIT1-based RNA purification effectively depletes rRNA and tRNA and produces high quality RNA-seq data. Moreover, we are reporting on artifacts generated by the use of RM showing depletion of some mRNAs, whereas such an artifact was not observed for the IFIT-based approach. Importantly, eIF4E(K119A) or yeast cap-binding proteins failed to enrich yeast mRNA, leaving IFIT1 as the only viable tool for 5′ end-dependent RNA capture in this species that can effectively replace eIF4E in such applications. Due to the high specificity of IFIT1 for cap 0, we believe that our method could be particularly useful for transcriptomic analysis in model and non-model lower eukaryotes or those relying on template-switching reverse transcription (22). We show that an IFIT-based approach can serve as a robust substitute method to commercially available approaches.

## MATERIALS AND METHODS

### Cdc33 and Cbp80-Cbp20 cloning

cDNA obtained with SuperScript^TM^ IV Reverse Transcriptase from total RNA isolated from *S. cerevisiae* BY4741 strain was used as a template to amplify ORFs coding for the full-length yeast Cdc33 (YOL139C; eIF4E), Cbp80 (YMR125W; STO1; CBC1) and Cbp20 (YPL178W; CBC2) in PCR with primer pairs: CDC33_F1 and CDC33_R1, CBP80_F1 and CBP80_R1, CBP20_F2 and CBP20_R2, respectively (0.2 μM of each primer, Supplementary Table S1), and using Phusion High-Fidelity DNA Polymerase (F530, Thermo Fisher Scientific) with 1 × HF buffer, 0.2 mM dNTP Mix and 3% DMSO. PCR products were gel-purified using Gel-Out kit (023-50, A&A Biotechnology). In the case of Cbp80 and Cbp20, second-round amplification was performed, using a mixture of the first-round PCR products as a template. Initially, 10 cycles of PCR product joining in the absence of primers was performed, based on the complementarity between fragments of CBP_R1 and CBP_F2 primers, followed by addition of CBP_F1-CBP_R2 primer pair and normal PCR, which eventually resulted in synthesis of the product corresponding to two-piece CBP80-CBP20 operon, which was purified as above. Final PCR products encompassing Cdc33 ORF and Cbp80-Cbp20 operon were inserted by SLIC into *Bam*HI/*Xho*I sites of pET28M N-6xHis-SUMO vector (23). *Escherichia coli* MH1 strain (*E. coli araD lacX74 galU hsdR hsdM rpsL*) was transformed with SLIC products. Positive clones were selected in standard LB medium containing kanamycin (100 μg/ml) and recombinant plasmids isolated with the use of Plasmid Mini kit (020-250, A&A Biotechnology) were validated by digestion with restriction endonucleases and Sanger sequencing.

### Recombinant Cdc33 and Cbp80-Cbp20 production and purification

*E. coli* BL21-CodonPlus(DE3)-RIL strain (Agilent; *E. coli* B F^–^*ompT hsdS*[r_B_^–^ m_B_^–^] *dcm*^+^ Tet^r^*gal λ[DE3] endA* Hte [*argU ileY leuW* Cam^r^]) was transformed with pET28M N-6xHis-SUMO vector derivatives carrying Ccd33 or Cbp80-Cbp20 inserts. Transformants were grown in a standard Luria-Broth (LB) medium supplemented with 50 μg/ml kanamycin and 34 μg/ml chloramphenicol overnight. Subsequently, 1 l of Auto Induction Medium (AIM) Super Broth Base including Trace elements (AIMSB02, Formedium) containing 2% glycerol and both antibiotics, was inoculated with 30 ml of the starter culture. Bacteria were grown for 72 h at 18°C with shaking (150 rpm) and eventually collected by centrifugation at 5000 rpm in a Sorvall H6000A/HBB6 swinging-bucket rotor for 15 min at 4°C.

Bacterial pellet was resuspended in 70 ml of lysis buffer (50 mM Tris–HCl pH 8.0, 200 mM NaCl, 10 mM imidazole, 10 mM 2-mercaptoethanol, 1 mM phenylmethylsulfonyl fluoride (PMSF), 0.02 μM pepstatinA, 0.02 μg/ml chymostatin, 0.006 μM leupeptin, 20 μM benzamidine hydrochloride), incubated with lysozyme (50 μg/ml; Roth) for 30 min in a cold cabinet, and then broken in an EmulsiFlex-C3 High Pressure homogenizer at 1500 Bar. The homogenate was centrifuged in a Sorvall WX Ultra Series ultracentrifuge (F37L rotor) at 33 000 rpm for 45 min at 4°C.

The supernatant was used for protein purification using the ÄKTA Xpress system (GE Healthcare), employing nickel affinity chromatography on an ÄKTA-compatible 5 ml column that was manually filled with Ni-NTA Superflow resin (Qiagen). The column was equilibrated with 25 ml of low-salt (LS) buffer (50 mM Tris–HCl pH 7.4, 200 mM NaCl, 10 mM imidazole, 10 mM 2-mercaptoethanol) prior to extract loading. After protein binding, the resin was sequentially washed with 40 ml of LS buffer, 25 ml of high-salt (HS) buffer (50 mM Tris–HCl pH 7.4, 1 M NaCl, 10 mM imidazole 10 mM 2-mercaptoethanol), and again with 20 ml of LS buffer. Bound proteins were recovered by elution with 30 ml of buffer E (50 mM Tris–HCl pH 7.4, 200 mM NaCl, 300 mM imidazole). Pooled eluate fractions (approximately 5 ml) were directly used for coupling with appropriate resins or optionally dialyzed overnight at 4°C against 2 l of LS buffer in the presence of 50 μg of home-made SUMO protease. In the latter case, the mixture was afterwards subjected to second round of purification on the nickel resin, performed using ÄKTA Purifier system (GE Healthcare) and employing LS buffer for collection of the flow-through, containing protein of interest devoid of the tag, and buffer E2 (50 mM Tris–HCl pH 8.0, 1 M NaCl, 300 mM imidazole) for elution of 6xHis-tagged SUMO or TEV protease and cleaved-off 6xHis-SUMOTag. Further purification of epitope-containing or cleaved-off target proteins from contaminating chaperones and degradation products was achieved by separation of the eluate from the first-round of affinity chromatography (no SUMO protease cleavage) or pooled flow-through obtained in the second-round of affinity chromatography (SUMO protease cleavage step included) on size exclusion Superdex 75 10/300 GL (Cdc33) or Superdex 200 10/300 GL (Cbp80-Cbp20) column (GE Healthcare) using 1.2 column volumes of gel-filtration (GF) buffer (0.1 M NaHCO3, pH 8.3 containing 0.5 M NaCl, or PBS with 0.5 M NaCl). Fractions corresponding to the maximum of A_280_ nm absorbance were collected after gel-filtration and pooled together for coupling with the appropriate resin.

### Preparation of home-made resins with coupled Cdc33 or Cbp80-Cbp20

Recombinant Cdc33 and Cbp80-Cbp20 dimer lacking or containing N-6xHis-SUMO tag were coupled to CNBr-activated SepFast MAG resin (BioToolomics; 310201–10G), following the manufacturer's instructions. Coupling of Cdc33 and Cbp80-Cbp20 to Dynabeads™ His-Tag Isolation & Pulldown magnetic beads (Invitrogen; 10103D) was carried out according to the manufacturer's recommendations, except for the omission of the elution step.

### Cloning, expression, and purification of human proteins

The human IFIT1 gene (identical with GenBank AK314588.1) was previously sub-cloned into the prokaryotic expression vector pETG10a (14) and expressed in BL21-CodonPlus(DE3)-RIL cells. Expression was carried out in LB medium, induced with 0.2 mM isopropyl-d-1-thiogalactopyranoside solution (IPTG) at OD600 of 0.6 and conducted overnight at 25ºC with shaking at 200 rpm. The cells were harvested by centrifugation at 4,000 × g, at 4°C for 20 min. The cell pellet was resuspended in buffer containing: 50 mM Tris pH 7.5, 0.5 M NaCl, 20 mM imidazole, 10% glycerol, 0.5 mM Tris(2-carboxyethyl)phosphine hydrochloride (TCEP), Complete protease inhibitor cocktail (Roche Life Science), DNase I and lysozyme. The suspension was lysed by sonication and the cell debris was removed by centrifugation at 48 880 × g for 30 min. The supernatant was applied on a HisTrap HP column (GE Healthcare) equilibrated in the same buffer. IFIT1 protein was eluted from the HisTrap HP column with a gradient of 20 mM to 500 mM imidazole in the buffer. The fractions containing purified protein were combined and diluted 5× with the buffer containing: 50 mM Tris pH 7.5, 10% glycerol, 0.5 mM TCEP. IFIT1 was next purified by heparin affinity using a HiTrap Heparin HP column (GE Healthcare), pre-equilibrated in buffer containing: 50 mM Tris pH 7.5, 100 mM NaCl, 10% glycerol, 0.5 mM TCEP. The protein was eluted with a gradient of 100 mM to 1 M NaCl in the buffer. Finally, IFIT1 was purified by size exclusion chromatography on a Superdex 200 Increase column (GE Healthcare Life Sciences) pre-equilibrated in 50 mM Tris pH 7.5, 150 mM NaCl, 5% glycerol, 0.5 mM TCEP buffer. The eluted protein samples were aliquoted and flash frozen in liquid nitrogen, and kept at –80ºC until use.

IFIT1 protein used for microscale thermophoresis was prepared by subcloning the IFIT1 gene from pETG10A_IFIT1 into the pET28a_His-SUMO-IFIT1 vector. IFIT1 protein was expressed and purified from BL21-CodonPlus(DE3)-RIL cells as described above with some modifications: after purification on a HiTrap Heparin HP column, His-SUMO tag was removed by incubation with SUMO protease with dialysis into 50 mM Tris–HCl pH 8.0, 150 mM NaCl, 1 mM DTT, 10% glycerol buffer and size exclusion chromatography was performed in PBS, 5% glycerol, 0.5 mM TCEP buffer.

GST-eIF4E(K119A) was purified following expression autoinduction in *E. coli* BL21-CodonPlus(DE3)-RIL strain by affinity chromatography on Glutathione Sepharose 4B resin and ion-exchange on ResourceS column, as described previously (10).

### *In vitro* RNA synthesis and 5′ end modification

The 4 different RNA molecules (80-mer, 100-mer, 135-mer, 160-mer) were 5′-terminal fragments of a sequence antisense to 7SK (7SK-as) of 80 nt, 100 nt, 135 nt and 160 nt respectively (Supplementary Table S2). The secondary structure of each RNA was predicted using RNAfold (ViennaRNA Web Service) to ensure that it forms a single-stranded 5′ end, required for binding by IFIT1. The vector with the 7SK-as construct was a gift from Giulio Superti-Furga (14).

The RNAs were transcribed *in vitro* from PCR-amplified templates using the HiScribe T7 High Yield RNA Synthesis Kit (New England BioLabs), according to the manufacturer's protocol. The RNA intended for microscale thermophoresis (Cap 0–100-mer) was body-labeled with Cy5 by the addition of 0.75 mM Cy5-UTP (Cytiva) to the transcription reaction. Obtained RNAs were loaded on a 5% (or 10% for Cy5-labeled RNA) polyacrylamide (PAA) gel containing 8 M urea and resolved in 0.5x Tris-borate-EDTA (TBE) buffer at 450 V for 4.5 h. The separated RNA samples were visualized using a UV imager. RNA-containing bands were excised from the gel and the Elutrap Electroelution system (Whatman) was used to purify RNA in 1× TBE at 100 V at 4ºC overnight. Cy5-labeled RNA was purified with Zymoclean Gel RNA Recovery Kit (Zymo Research) after excision from the gel.

The purified 5′ triphosphate RNA 135-mer was left unmodified (as the original product of IVT reaction). Other purified RNAs were further modified enzymatically on the 5′ ends according to the enzyme manufacturer's protocols. The 80-mer was dephosphorylated with alkaline phosphatase (Calf Intestinal Phosphatase, New England BioLabs) in order to obtain 5′OH-RNA. The 100-mer was treated with RNA 5′ pyrophosphatase (RppH, New England BioLabs) to obtain 5′p-RNA of 100 nt. The Vaccinia Capping System (New England BioLabs) was used to generate the 5′cap 0-RNA of 160 nt (160mer) or 100 nt (Cap 0–100-mer, used for microscale thermophoresis). The modified RNA was purified with Rneasy Mini Kit (Qiagen) or in the case of Cap 0–100-mer with Monarch® RNA Cleanup Kit (New England BioLabs). The 4 RNA mixture for a pull-down reaction was prepared by combining 70 picomoles of each RNA (80-mer, 100-mer, 135-mer, 160-mer).

### RNA extraction

RNA was extracted from the *S. cerevisiae* strain BY4741, using a published protocol (24) with modifications. 35 ml yeast cultures were grown in YPD (1% yeast extract, 2% peptone, 2% dextrose) to an OD600 of 0.6. The cells were harvested by centrifugation and the pellet was resuspended in 500 μl of AE Buffer (50 mM NaOAc pH 5.3, 10 mM EDTA), supplemented with 10% SDS. The suspension was vortexed with an equal volume of fresh phenol, previously equilibrated with an AE buffer. The mixture was incubated at 65°C for 5 min, rapidly chilled in liquid nitrogen, thawed at room temperature and centrifuged for 20 min at maximum speed to separate the aqueous and phenol phases. The aqueous phase was extracted twice with an equal volume of phenol/chloroform and once with $\frac{1}{2}$ vol. of chloroform. RNA was precipitated from an aqueous phase by adding 1/10 vol. of 3 M NaOAc pH 5.3 and 2.5 vol. of ethanol, washed twice with 1 ml of ice cold 75–80% ethanol. RNA was submitted to treatment with TURBO DNase (Thermo Fisher Scientific) and RNA clean-up (with RNeasy Mini Kit, Qiagen) according to the manufacturer's protocols. RNA was aliquoted and stored at -80°C.

For pull-downs of RNAs using eIF4E(K119A), Cbp20/80 or Cdc33, yeast cell pellets were resuspended in 400 μl of TES buffer (10 mM Tris pH 7,5; 5 mM EDTA; 1% SDS), supplemented with 400 μl of phenol solution saturated with 0,1 M citrate at pH 4.3 (Sigma-Aldrich P4682) and vortexed for 40 and then 20 minutes at 65°C. Between incubations samples were centrifuged at 14 krpm for 10 min at 4°C. The aqueous phase was washed with 400 μl of chloroform, centrifuged as previously and precipitated with 45 μl of 2 M LiCl in 1 ml of 96% ethanol at -80°C for at least 30 min. RNAs were pelleted, washed for 15 min with 80% ethanol, dried and resuspended in RNase free water. Human RNAs were extracted by resuspending cell pellets in 1 ml of Tri Reagent (Sigma-Aldrich T9424) and incubating for 5 min at room temperature. Subsequently 0.2 ml of chloroform was added and the samples were vortexed for 3 minutes and incubated for 10 min at room temperature. The samples were centrifuged for 15 min at 14 krpm at 4°C and the aqueous phase was precipitated with 0.5 ml of isopropanol. The pellets were washed with 75% ethanol, dried and resuspended in water for further use.

### RNA capture by pull-down using IFIT1

Two alternative pull-down protocols were used, ‘Complex on Beads’ Con-B (Supplementary Box S1) and ‘Complex in Solution’ Cin-S (Supplementary Box S2). In the Con-B method, a 50 μl portion of the Ni^2+^-Sepharose 6 Fast Flow (GE Healthcare) slurry beads was washed with RNase-free water and equilibrated in a chilled Binding Buffer (50 mM Tris pH 7.5, 150 mM NaCl, 1 mM DTT, 5 mM imidazole, 0.01% Tween 20, 3 mM MgCl_2_). Beads were sedimented by centrifugation at 500 × g for 1 min at 4°C and the supernatant was discarded. A portion of 2–3 μg (35–55 pmol) of 6xHis-IFIT1 was immobilized on the beads under rolling mixing for 0.5–1 h at 4°C. The beads were sedimented as before and washed with 1 ml of the Binding Buffer. The binding of IFIT1 with RNA (a mixture of 4 of the *in vitro* transcribed RNAs (Supplementary Table S2) or 10 μg of the total yeast RNA) was performed in 1 ml of Binding Buffer supplemented with poly(dI-dC) (Poly(deoxyinosinic-deoxycytidylic acid) sodium salt, Sigma-Aldrich) at a final working concentration of 2 μg/ml, under rolling mixing for 1 h at 4°C. Prior to the capture procedure, RNA samples were heat denatured at 65–70°C for 10 min and placed on ice for 5 min before the binding. In the Cin-S method, IFIT1 was bound with RNA in 1 ml of the Binding Buffer supplemented with Poly(dI-dC) under rolling mixing for 1 h at 4°C and after that, the mixture was transferred to the pre-equilibrated beads for IFIT1-RNA complex immobilization. After binding, the beads were sedimented as before and washed several times with 1 ml of chilled Wash Buffer (50 mM Tris pH 7.5, 250 mM NaCl, 1 mM DTT, 5 mM imidazole, 0.01% Tween 20, 3 mM MgCl_2_). Selected RNA was released by proteolysis with Proteinase K (Invitrogen) for 1 h at 37°C. The eluted RNA was precipitated with ethanol, re-suspended in RNase-free water and used for further reactions. RNeasy Mini Kit (Qiagen) was used for RNA clean-up prior to RNA-seq.

Yeast RNA samples (total RNA and RNA recovered after the pull-down) were analyzed by high-resolution automated electrophoresis with Agilent Bioanalyzer 2100 and Agilent RNA 6000 Pico Kit (according to the manufacturer's protocol).

RNA oligonucleotides recovered after the pull-down (Con-B protocol) of the *in vitro* transcribed mix of the four RNAs were separated in a 12% PAA gel containing 8 M urea, stained with SYBR Gold Nucleic Acid Gel Stain (Thermo Fisher Scientific) and visualized on a ChemiDoc MP Imaging System (Bio-Rad).

### RNA capture using human eIF4E(K119A) or yeast Cdc33 or Cbp80-20

50 μl of MagneGST magnetic beads (Promega V8600) per 1 sample were washed 3 times with 1 ml of PBS. The GST binding capacity declared by the manufacturer at 1 mg and is far thus below the amounts of GST-eIF4E(K119A) that were subsequently applied to the resin (0, 0.015, 0.03, 0.06 and 0.090 mg) and bound for 1 h in PBS at 4°C on a rotating wheel. 200 μl of CNBr-activated SepFast MAG resin with coupled Cdc33 or Cbp80-20 dimer (encompassing or lacking N-6xHis-SUMO tag) or 40 μl of Dynabeads™ His-Tag Isolation & Pulldown magnetic beads with bound N-6xHis-SUMO tagged Cdc33 or Cbp80/20 were used in parallel, at approximately 0.1 mg protein/ml. The resins were washed 3 times in 1 ml PBS and then 3 times in 1 ml IP buffer (10 mM potassium phosphate buffer, pH 8.0, 100 mM KCl, 2mM EDTA, 5% glicerol, 0.005% Triton X-100, before use 6mM DTT and 20U/ml Ribolock was added). 120 μg of total RNA in 50–90 μl water were denatured at 70°C for 10 min and cooled for 2 min on ice, The RNAs were supplemented to 500 μl with IP buffer and incubated with the MagneGST-eIF4E(K119A) resin for 90 minutes at 4°C on a rotating wheel. The flow-through was collected for control analyses. The resin was washed 3 times with 1 ml of IP buffer, 2 times with IP buffer containing 0.5 mM GDP, which should reduce background levels, and again 2 times with 1 ml of IP buffer. The resin was resuspended in 500 μl of IP buffer for elution. Elution was performed with the protocol for RNA extraction using 400 μl of phenol solution saturated with 0.1 M citrate at pH 4.3 and following all the steps described above.

### Sequencing and NGS data analysis

Depletion of rRNA with a commercial RiboMinus™ Transcriptome Isolation Kit, yeast (Invitrogen) was performed according to the manufacturer's protocol. Samples for small-scale trial sequencing were sent to Eurofins Genomics, where polyA-based enrichment was performed according to the Standard eukaryotic library service and compared with our Con-B and RiboMinus ribodepleted samples. mRNA was fragmented and cDNA synthesis was performed using random hexamer priming, and analyzed by Illumina NovaSeq 6000 sequencing using 150 bp paired-end reads.

**Figure 2.**
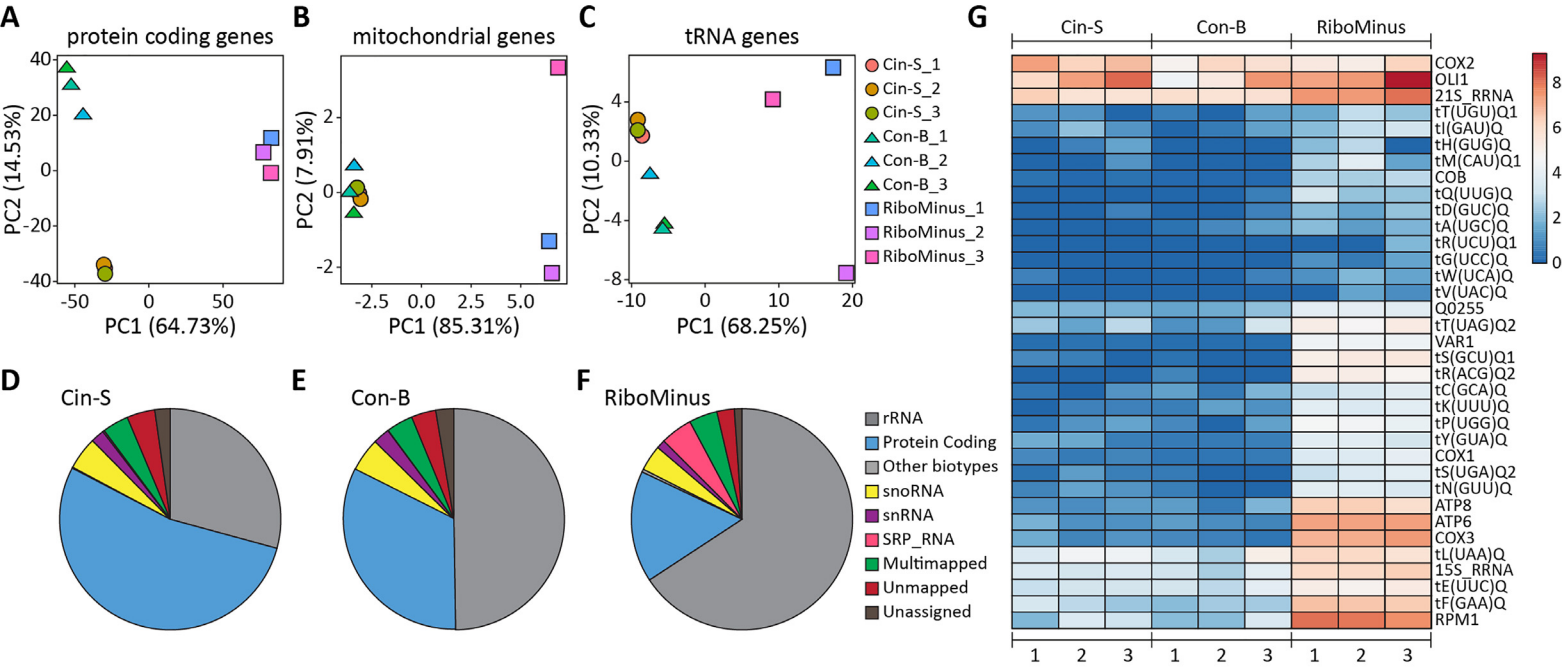
Comparison of RiboMinus (RM) and IFIT1-based approaches (Cin-S, Con-B) for mRNA enrichment. Principal component analysis (PCA) for (**A**) protein coding genes, (**B**) mitochondrial genes, (**C**) genes encoding tRNAs. Biotypes of (**D**) Cin-S, (**E**) Con-B and (**F**) RM datasets. (**G**) Heatmap representing mitochondrial transcripts (log_2_(TPM + 1)).

**Figure 3.**
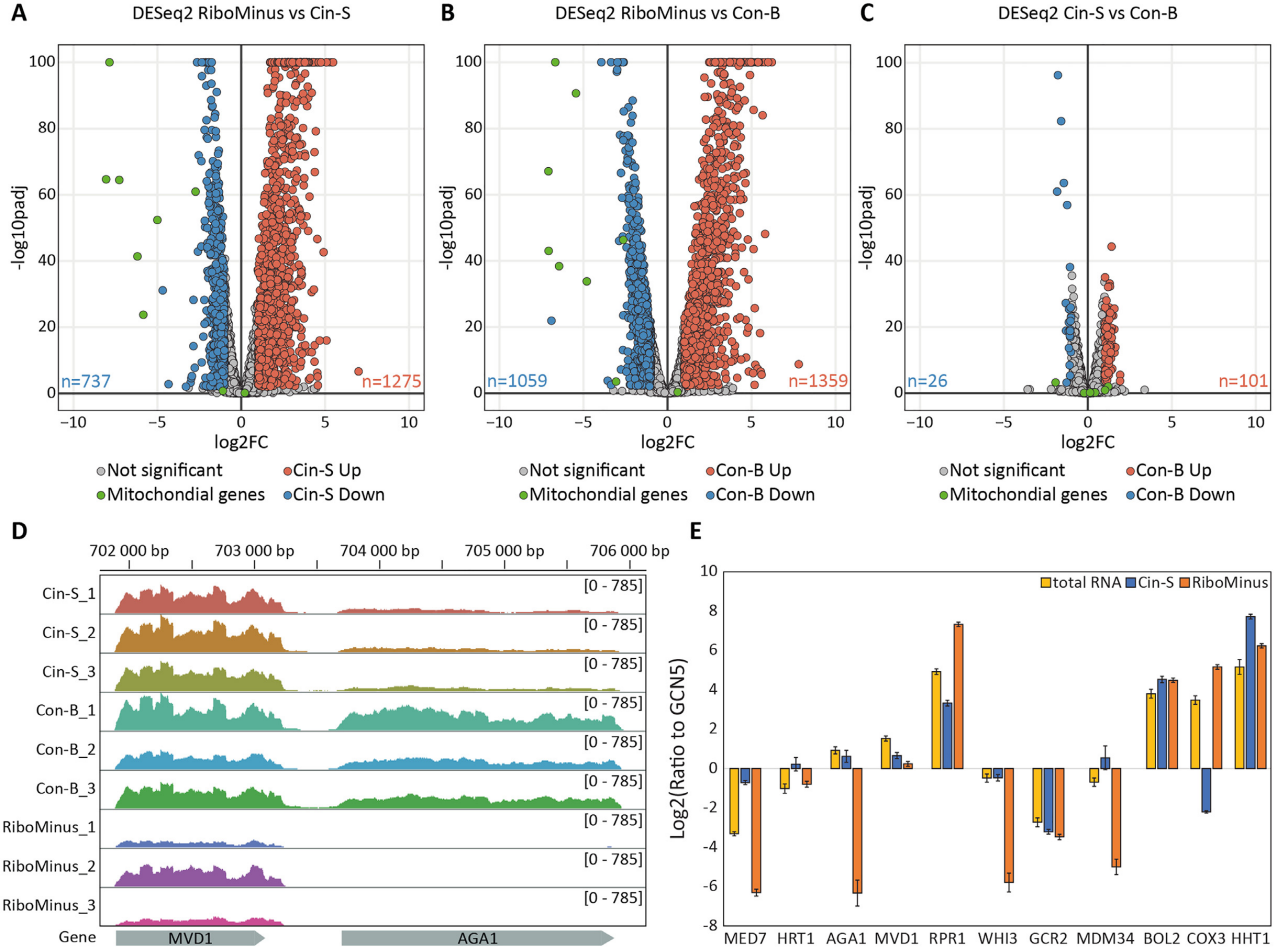
Differential analysis of count data (DESeq2) for pairwise comparisons of RM with Cin-S **(A**), RM with Con-B (**B**), and Cin-S with Con-B (**C**) datasets. Statistically significant transcripts (*P*_adj_ ≤ 0.01) with at least 2-fold change (FC) were marked in blue (respective decrease) or red (respective increase), and mitochondrial transcripts were highlighted in green; *P*_adj_ – Benjamini–Hochberg adjusted *P*-value. At the bottom of the graphs on the left and right side are the numbers of transcripts with significantly decreased and increased enrichment, respectively. (**D**) Plots representing reads mapped to the AGA1 and MVD1 genes. (**E**) RT-qPCR quantification of MED7, HRT1, AGA1, MVD1, RPR1, WHI3, GCR2, MDM34, BOL2, COX3 and HHT1 relative ratios to the GCN5 reference gene.

Samples for large-scale sequencing were prepared in triplicates for Cin-S, Con-B and RiboMinus ribodepleted samples. The strand-specific cDNA library was prepared using an Illumina Tru-Seq kit by the Genomics Core Facility, Centre of New Technologies, University of Warsaw. Sequencing was performed using Genome Sequencer Illumina NovaSeq 6000 PE100. Sequencing of nine samples generated 406 681 674 reads in two paired-end fastq files per experiment. Raw sequences have been deposited in ENA: PRJEB49214, the analyzed data and description of the samples were deposited in GEO: GSE210198. The quality of the data received from the sequencing was checked with FastQC v0.11.9 software. Reads were trimmed and filtered to remove contaminations and low quality reads with Trim Galore v0.6.7 (–quality 20, default auto-detection of adapters; Supplementary Tables S3–S4). The high-quality filtered paired-end reads were aligned to *Saccharomyces cerevisiae* rRNA with bowtie2 v2.4.4, then resulting sequences were counted and removed from further analysis. All remaining reads were aligned to the genome of *S. cerevisiae* (NCBI: GCF_000146045.2_R64) using STAR v2.7.9a, allowing up to 4% of mismatches per read length (Supplementary Table S5). The resulting BAM files were used in the IGV v2.11.4 program to generate coverage plots of genes (Figure 3D; Supplementary Figures S9 and S10). In the next step, all uniquely mapped reads were counted using featureCounts v2.0.3 and normalized to TPM (transcript per million) either for all reads or for reads mapping to coding sequences only, using a custom R script (Supplementary Data 1; Supplementary Table S6). Using TPM data, we generated bar charts and heatmap of expression for selected genes (Figure 2G; Supplementary Figure S8). Based on the annotations from the NCBI database and including previously deleted rRNA sequences, the percentages of individual RNA biotypes for all experimental groups were calculated (Figure 2D–F; Supplementary Tables S7–S8). For the rRNA heatmap (Supplementary figure S5A), sequences removed at the bowtie2 filtering stage were also included. The Venn diagram of protein-coding genes (Supplementary figure S5B) detected by each method was generated with InteractiVenn (25) from genes having at least 10 raw reads in each replicate. The raw counts without non-coding sequences were loaded to the DEBrowser v1.22.2 web tool and genes with a maximum number of raw reads of 10 or less in all samples were filtered out. For PCA and volcano plots of differential expressed genes, the data was normalized with MRN (Supplementary Data 2: median ratio normalization; Figure 2A–C; Figure 3A–C; Supplementary Figure S3A–C). Differential expression analysis was performed with DEBrowser v1.22.2 using the DESeq2 v1.34.0 library (Fit Type:Parametric; Test Type:Wald; Supplementary Data 3).

### Analyses of the 5′ UTRs of mRNAs

The first 10 nucleotides of the 5′UTR for coding RNAs were extracted from the genome, based on the main promoter downloaded from the EPDnew database (26). The sequences were used to generate WebLogos (WebLogo 3.7.11 (27,28). We have analyzed 4920 UTRs out of 5794, all for which sequences were annotated. UTRs of the genes having internal transcription start site were not included. For analysis of IFIT1 sequence preference, we counted the prevalence of the given base in each position, in the groups of mRNA that were defined by the intervals of differences in counts (fold change) in Cin-S or Con-B as compared to RM. The results were plotted with Microsoft Excel as percent of the given base in the mRNA group.

### Reverse transcription and real-time PCR

After pull-down experiments, 70 ng of recovered RNA was subjected to a reverse transcription (RT) reaction using First Strand cDNA Synthesis Kit (Roche Life Science) and random hexamers, according to the manufacturer's protocol). The resulting cDNA was used for real-time quantitative PCR (qPCR). qPCR was performed in a final volume of 25 μl containing 1x HOT FIREPol EvaGreen qPCR Mix Plus (ROX) (Solis BioDyne), 0.8 μM forward primer and 0.8 μM reverse primer, according to the manufacturer's protocol. Three technical replicates were performed for each qPCR reaction. Primers used for the qPCR are listed in the Supplementary material (Supplementary Table S9). Thermocycling reactions were performed using a LightCycler 96 Instrument (Roche Life Science), with an initial denaturation step at 95°C for 5 min, followed by 40 cycles of 15 s at 95°C, 20 s at 60°C and 20 s at 72°C. The results were analyzed by the relative quantification using software provided by the manufacturer (Roche Life Science) and experimentally determined primer efficiency coefficients, comparing the level of selected yeast transcripts: MED7, HRT1, AGA1, MVD1, RPR1, WHI3, GCR2, MDM34, BOL2, COX3, HHT1, 18S rRNA, TDH3, RPS13, RPL21B, RPL28 and RPL36A) or human transcripts (GAPDH, NOSIP, WDR61, CTR9, SKIV2L and TTC37) (Supplementary Table S9). NCBI’s BLAST (Basic Local Alignment Search Tool) was used to design target-specific primers and avoid off-target amplification. The enrichment of the material with selected RNA molecules was then determined by comparing the relative amount either before or after the pull-down procedure to the reference gene (GCN5) or displayed as change relative to the input. The reference gene was selected based on NGS results and a constant copy number in all analyzed samples. A no reverse transcriptase control (NRT) assessment during qPCR demonstrated no DNA contamination of RNA samples. All total RNA samples had a RIN value above 7.8. A RIN value was not applicable to pull-down samples due to rRNA depletion.

### Microscale thermophoresis

MST experiments were performed on a Monolith NT.115 system (Nanotemper Technologies) at 22°C with 60% IR-laser power and 60% LED in MST buffer (PBS, 5% glycerol, 0.5 mM TCEP, 0.05% Tween20) using premium capillaries. The concentration of Cy5-labeled cap 0–100-mer RNA was kept constant at 10 nM. Unlabeled protein was titrated in 1:1 dilutions with the highest concentration of 1 μM IFIT1 or 50 μM eIF4E(K119A). The recorded fluorescence was normalized to the fraction bound and analyzed using GraphPad Prism 9.3.0.

## RESULTS

### eIF4E pull-down can enrich human, but not yeast mRNA

Cap 0 is commonly found in budding yeast *Saccharomyces cerevisiae* but is rare for human RNAs (Figure 1A), making a cap 0-based mRNA purification protocol potentially of great interest to the yeast community, for example in studies of poly(A) tails (29). Purification of mRNAs for transcriptomic analyses using eIF(K119A) was developed for human and mouse studies, and mostly demonstrated in vertebrates which have cap 1 or higher-methylated mRNA caps. Therefore, to test the utility of eIF4E(K119A)-based protocol towards yeast mRNAs we compared the efficiency of yeast and human mRNA enrichment using increasing amounts of eIF4E(K119A). We noted that the absolute level of human, but not yeast, RNA bound was proportional to the amount of eIF4E(K119A) used as the bait (Supplementary Figure S1A). Consistently, reverse-transcription coupled to real-time quantitative PCR (qPCR) analysis of a selected pool of transcripts showed no specific enrichment of yeast mRNAs, in contrast with a maximum average 50% mRNA purification efficiency of human coding transcripts, which was attained at average bait amounts (Supplementary Figure S1B). Indeed, inspection of the RNA quality on agarose gel suggested that the increased amounts of RNA purified at a higher eIF4E(K119A) concentration was likely due to non-specific binding to ribosomal RNA (Supplementary Figure S1C), providing a highly contaminated sample when used by inexperienced hands. Although the K119 residue mutated in human eIF4E, which increases the protein's affinity towards capped RNA, is not conserved in yeast, we next attempted to use also yeast cap-binding proteins for mRNA enrichment. We purified the yeast eIF4E homolog Cdc33 along with the nuclear Cbp20/80 cap binding complex and assayed its binding in various experimental settings to yeast total RNA and saw no substantial enrichment (Supplementary Figure S1D, E). To our knowledge, no published or commercial protocol exists for purification of yeast mRNAs via the cap, and we thus next pursued development of our new IFIT1-based method.

### IFIT1 selectively and efficiently binds RNA molecules in a cap 0-dependent manner

We experimentally evaluated and optimized RNA pull-down conditions for IFIT1 using a small pool of artificial RNAs with various 5′ ends (Figure 1C; Supplementary Table S2). We mixed RNAs with 5′ cap 0, ppp, p and OH modifications representing four possible RNA forms found in the yeast cell (Figure 1D): mature cap 0 mRNA, triphosphorylated RNAs which have not yet been subjected to a capping reaction or processing; monophosphorylated RNAs which are products of exo- or endo-nucleolytic cleavage, and hydroxylated RNAs which arise mainly from chemical cleavage. His-tagged IFIT1 immobilized on Ni-sepharose resin efficiently enriched samples for cap 0-modified RNA (Figure 1C), with an excellent affinity for cap 0-RNA (apparent *K*_D_ = 47 ± 2.4 nM) (Supplementary Figure S2).

This high selectivity of IFIT1 towards RNAs modified with cap 0 allowed us to apply an IFIT1-based pull-down to much more complex samples to achieve mRNA enrichment prior to a transcriptomic analysis. To this end, we isolated total RNA from yeast and captured its cap 0 fraction in two alternative approaches (Supplementary Box S1 and S2). To optimize IFIT1 binding with RNA, we introduced the Cin-S (‘Complex in Solution’) capture method (Figure 1E; Supplementary Box S2) that allowed protein-RNA complex formation prior to immobilization on the resin via the affinity tag of IFIT1. For comparison, we also used the initial Con-B (‘Complex on Beads’) approach (Figure 1E; Supplementary Box S1) in which IFIT1 was attached via affinity tag to the resin prior to binding with RNA. The Cin-S method was intended to enable more accessibility and flexibility of IFIT1, whereas Con-B was thought to promote preferential orientation of N-terminal tags towards the resin and therefore exposure of the C-terminal RNA-binding sites of IFIT1 to the solution. In each case, washing steps removed the unbound, cap-less RNA from the RNA-IFIT1 complexes immobilized on the resin, and in the final extraction step using Proteinase K digestion the cap 0-RNA could be easily recovered for use in downstream NGS analyses. We successfully prepared RNA samples processed according to both Cin-S and Con-B protocols for the comparison of their performance in RNA-seq library preparation.

### IFIT1-based RNA selection is a viable method for RNA library preparation

In order to identify the right benchmarking method, we started our NGS analyses with a small-scale trial experiment comparing two commercially available ribodepletion approaches, RiboMinus (RM) and polyA enrichment, with the Con-B approach applied to the total yeast RNA (raw data not included). The Principal Component Analysis (PCA) for RM, polyA and Con-B showed PC1 and PC2 contributions of 63% and 20.41%, respectively (Supplementary Figure S3A, B). The polyA-enriched dataset was distinctly located more distant from other datasets (PC1), while RM and IFIT1-based approaches seemed more closely related. This result confirmed our expectations that the 3′ end-dependent method might differ substantially from our 5′ end-dependent IFIT1 pull-down, as it is known also to vary from eIF4E capture (9), other custom ribodepletion methods (30) and commercial ribodepletion results (4,5,31). We therefore next focused on the use of RM as the commercially available benchmark method for a large-scale evaluation of our IFIT1-based pull-down approach.

We compared the RNA samples recovered from the IFIT1 pull-down (Cin-S and Con-B) with those obtained using the commercially available RiboMinus (RM) standard ribodepletion procedure applied to total yeast RNA. Triplicates for each approach were prepared and sequenced using Illumina NovaSeq 6000 PE100. We received 406.7 million paired-end raw reads in total (ranging from 25.6M to 88.9M reads per sample) with a mean length of the read of 101 bp. In the first stage, data was trimmed and filtered to remove low-quality reads. On average, about 38% of sequences contained adapters. After adapters removal and verification of the data quality (mean Phred quality score min. 20), 6.808 to 76.621 sequences per sample were deleted (Supplementary Table S3). In the following step, sequences were mapped to the rRNA reference fasta file generated from the reference genome and the GTF annotation file, and 7.5M to 60.4M sequences were removed from further analysis (28–68% of all sequences in samples; Supplementary Table S4). The remaining reads were mapped to the yeast reference genome (NCBI: GCF_000146045.2_R64). Approximately, 7.8M to 33.7M reads were uniquely mapped to the genome (79.9–89.6%; Supplementary Table S5) and then counted. We received 7.4M to 31.1M reads assigned to the genes (Supplementary Table S6; Supplementary Data 1). At all stages of data analysis, the statistics (mapped and uniquely assigned reads) we obtained for Cin-S and Con-B methods were comparable with those resulting from the standard RM approach. In all Principal Component (PC) analyses, the triplicates within Cin-S and Con-B datasets grouped closely, indicating good reproducibility (Figure 2; Supplementary Figure S3C; Supplementary Data 2). Typically, RM derived data placed more distantly from the Con-B and Cin-S data (PC1), and the RM replicates exhibited more variability (PC2) for mitochondrial (Figure 2B) and tRNA (Figure 2C) genes. The mean coverage and read distribution for all genes was also comparable between Cin-S, Con-B and RM approaches, with the highest overall mean coverage for the Con-B method (Supplementary Figure S4A).

### IFIT1-based capture method selectively enriches nucleus-encoded mRNA

The number of reads in each biotype, including previously removed rRNA counts, were recalculated for each experimental group according to the reference data (Figure 2D-F; Supplementary Figure S5A; Supplementary Tables S7 and S8). This detailed biotyping showed decreased rRNA counts in Cin-S (29.23%) and Con-B (49.75%) datasets when compared to RM data (65.76%), with depletion efficiency in IFIT1 pull-downs especially pronounced for 5S and 5.8S rRNA and less for 18S and 25S rRNA (Supplementary Figure S5A). This improved rRNA depletion was accompanied by improved protein-coding RNA counts (53.42%, 32.65% and 16.27% for Cin-S, Con-B and RM, respectively) and enriched transcripts detection (Supplementary Figure S5B). We compared mRNA with at least 10 raw reads in our datasets (which criterion excluded ∼5%, i.e. 300 out of 6002 transcripts), and while the large majority (90%) of protein-coding RNAs was identified with such threshold for all three methods, an additional ∼4% (233 transcripts) was identified in both Cin-S and Con-B but not RM, and further 1% (57 transcripts) by only Cin-S or Con-B, whereas only ∼0.1% (6 transcripts encoding: mitochondrial ATP8 and VAR1, membrane proteins HXT6, VTH2 and YKR104W, and uncharacterised YOL013W-A) were found in RM alone but not in IFIT1 pull-downs (Supplementary Figure S5B). All datasets contained very little tRNA counts (0.1–0.2%). In addition, Cin-S and Con-B approaches recovered very little SRP RNA (0.25% and 0.04%, respectively), which contrastingly accounted for 4.68% of all mapped RNAs in RM data. Other non-coding transcripts which decreased in the Cin-S and Con-B samples included RPR1 (the component of nuclear RNase P, transcribed by RNA polymerase III), some snoRNAs (e.g. snR70, snR17a, snR17b, snR190) and snRNA (e.g. snR6, which is U6 snRNA, also a product of RNA polymerase III), but not all non-coding RNA were affected (e.g. snR41, snR86) and some were relatively enriched in IFIT1-based pull-downs (e.g. snR4, snR11 or snR19 which is U1 snRNA) (Supplementary Figure S6; Supplementary Data 1B). Among the non-coding RNAs, those with cap-less ends due to processed 5′ ends (the majority of C/D box snoRNA) or lack of capping (RNA polymerase III products) were relatively depleted in IFIT1 pull-downs, while enriched non-coding RNAs included capped transcripts such as H/ACA box snoRNA which typically receive trimethylated cap structures (m_3_^2,2,7^GTP) post-transcriptionally (32). The most striking differences in the case of mRNAs were seen for those originating from genes encoded within the mitochondrial genome (Figure 2B, G). In both Cin-S and Con-B datasets, we detected only low levels of the majority of mitochondrial transcripts, except for COX2 and OLI1 transcripts (Figure 2G).

### IFIT1-based transcriptome analysis avoids off-target hybridization artifacts

We next ascertained whether the IFIT1-based mRNA capture introduced any bias to the mRNA content in comparison with the original total RNA sample. As noted in the biotype analysis, Cin-S and Con-B data showed a decrease in some mitochondrial mRNA (e.g. ATP6, ATP8, COB, COX1, COX3, VAR1) and non-coding transcripts (RPM1, the RNA component of mitochondrial RNase P) as compared to RM (Figure 2G, Figure 3A-B, Supplementary Data 3). The Cin-S and Con-B approaches showed a very good correlation with each other (Supplementary Figure S7) and similar read counts per gene (Figure 3C). We observed that some transcripts had significantly lower read counts in the RM samples than in Con-B or Cin-S samples (Supplementary Figure S8). Among mRNAs that demonstrated low coverage in RM samples were MED7 and AGA1, whereas their neighboring genes HRT1 and MVD1, respectively, showed comparable coverage in all samples and methods (Figure 3D; Supplementary Figure S9). Since the mean coverage for all genes (Supplementary Figure S4) and the coverage of the housekeeping genes such as GCN5 (Supplementary Figure S10) did not indicate any problems with the general performance of the RM method and dataset (nor with Cin-S or Con-B), we attributed this selective difference to putative off-target depletion of some mRNAs such as MED7 and AGA1, most probably due to hybridization to RiboMinus probes during the ribodepletion step procedure.

We next validated these differences for selected genes using RT-qPCR on the total RNA, Cin-S and RM samples (Figure 3E; Supplementary Figure S11). We confirmed the efficient removal of rRNA in Cin-S by comparing 18S rRNA levels with the reference GCN5 mRNA (Supplementary Figure S11A). RT-qPCR results confirmed the selective decrease in mitochondrial transcripts such as COX3 in the Cin-S samples, and the relative depletion of MED7, AGA1, WHI3 and MDM34 in the RM samples (Figure 3E; Supplementary Figure S11B, C), whereas their neighboring genes (HRT1, MVD1, GCR2, BOL2, HHT1) showed comparable levels in both sample types. To summarize, apart from the expected cap-dependent differences in mitochondrial and non-coding transcripts, we did not detect any problems with mRNA recovery by the IFIT1-based capture method; conversely, our approach overcame artifacts due to the alternative RM method.

### Sequence specificity of the IFIT1 protein toward RNA

In the differential analysis of count data (Figure 3A, B), the IFIT1-based approach showed a slight bias in the enrichment of the mRNAs. We also observed a slight increase in 5′-proximal reads for Cin-S relative to RM in the region following the translation start site (TSS) in the coverage of the averaged metagene, whereas RM showed an increase in the region centered around ∼100–150 nt preceding the TSS (Supplementary Figure S4B). Possibly, some mRNA relatively depleted in IFIT1 pull-downs may be due to the presence of degradation or processing products lacking caps, for example we noted one of the most decreased count values (e.g. fold Change of 0.065 for Con-B in regard to RM, Supplementary Data 3) for the HAC1 transcript which contains known endonucleolytic cleavage sites (33). We tested this hypothesis by comparing our results with published studies of decapped intermediates either identified in the 5′-3′ co-translational decay (34) or stabilized in *xrn1-* strain (35). Decapped transcripts prone to degradation by the 5′ exonuclease Xrn1 identified in these studies did not show any significant correlation with the abundance of transcripts decreased in IFIT1 pull-downs nor in the RiboMinus dataset (Supplementary Figure S12, Supplementary Data 4). mRNAs relatively depleted in IFIT1 pull-downs were present both among transcripts with high and low codon protection index (CPI, a signature of regulation by the 5′-3′ co-translational decay). For example, YOL075C and FEN2 with high CPI, as well as HAC1 and YIR016W with low CPI (Supplementary Data 4), are all among the top 50 RNAs relatively depleted in both Cin-S and Con-B as compared to RM (Supplementary Data 3). The low CPI for HAC1 mRNA might be in line with an endonucleolytic initiation of degradation other than the co-translational exoribonucleolytic decay by Xrn1, and IFIT1 pull-downs might show decreased counts in both cases. The mRNA decay intermediates are less pronounced during yeast growth in the rich media such as YPD used in our experiments, as compared to stress conditions (the median of CPI is 0.536 in the YPD dataset of (34)), which might have contributed to their observed poor correlation with decreased enrichment on IFIT1 (the median of CPI in the set of transcripts with fC ≤0.5 in Cin-S relative to RM was 0.542, indicating low or no prevalence of co-translational degradation). Nonetheless, comparisons with Xrn1-dependent datasets could not let us attribute the bulk of the mRNAs relatively underrepresented in IFIT1 pull-downs to the presence of decapped intermediates. Moreover, the mRNA relatively enriched in Cin-S and Con-B should be attributed rather to preferential binding by IFIT1. We therefore looked at the trends in sequences of the yeast mRNA as ranked by their differential enrichment or depletion in IFIT1 pull-downs in comparison with RM. Since IFIT1 binds to the cap 0, and the largest RNA fragment found in the co-crystal structure of IFIT1 amounts to 10 nt Cap 0-RNA (Figure 1B) (18), we analyzed IFIT1 sequence specificity based on the first 10 nucleotides of the 5′UTR. Sequences of the UTRs were grouped based on the fold Change value compared to the RM data. We observed consistent differences at several UTR positions in both Cin-S and Con-B datasets (Supplementary Figure S13, Supplementary Data 3). Notably, with the exception of the third nucleotide, IFIT1 in general preferred adenines, which were slightly overrepresented in the most enriched mRNA group (Supplementary Figure S13B, D), but at the same time correspond to the general presence of adenine as the most prevalent base throughout the yeast UTRs (Supplementary Figure S13A). At the third, fourth and fifth positions, IFIT1 showed increased preference for cytosines, while guanines were slightly depleted, especially in the fourth position. However, none of these changes in enriched or depleted base identities exceeded more than about 10% of the average base frequency, and were typically only a few percent over- or under-represented in the UTRs of the relatively most enriched or depleted mRNAs (quantified percentages in Supplementary Figure S13). We could not identify any preferred sequence motif among mRNA preferentially enriched on IFIT1, as evidenced by the variability in the top enriched 5′ UTR sequences (for example, the top three transcripts in Cin-S vs. RM: TIM13, HSP150 and FYV5 with sequences 5′AAUCAAUCUU, 5′AUCAAUAAGA and 5′AGCCGGAUAU, respectively; Supplementary Data 3A). We conclude that IFIT1 shows a slight base composition preference which contributes to the differences in mRNA counts, but that IFIT1 binds robustly to all cap 0-RNA.

## DISCUSSION

mRNA enrichment using the cap-binding eIF4E protein is commonly used for transcriptomic analyses, especially those focusing on 3′ ends (10). Often, it is used in tandem with standard ribodepletion in order to improve sequence coverage of the coding transcriptome, since eIF4E(K119A) pull-downs of human mRNA retain a large proportion of 28S rRNA (10). In addition, cap-dependent capture is thought to enrich more mature, intact mRNAs as opposed to degradation intermediates that underwent decapping (33). It also differs from the Cap Analysis Gene Expression (CAGE) method which requires chemical oxidation and biotinylation of the cap for capture on streptavidin, which is typically combined with RNase digestion and thus yields 5′-proximal mRNA fragments in order to map transcriptional start sites (36). In contrast, protein-based mRNA capture via the cap is used rather for enrichment and studies of full-length transcripts.

Surprisingly, in our attempt with human or yeast eIF4E, its binding to yeast RNA did not perform as well as for another less known cap-binding protein, IFIT1. This might have resulted from unsuccessful purification or instability of eIF4E. However, since eIF4E(K119A), in our expert hands, was able to pull down human mRNA, we concluded that the quality of the protein sample was not the source of the poor binding to yeast mRNA. Nonetheless, problems with protein production or stability may also regard other protein affinity-based transcriptomic analyses, and it is worth noting that under the same experimental conditions, IFIT1 performed more robustly than eIF4E (nor could we find published studies of yeast mRNA capture by eIF4E). Another explanation is that poor capture efficiency by eIF4E may stem from the nature of 5′ ends of the analyzed RNA, for example due to obstructive or missing RNA modifications, such as defective N7-methylation of the cap. Unlike eIF4E, IFIT1 has no dependence on N7-methylation, which could facilitate mRNA capture in suboptimal or degraded samples (in which N7-methyl group may be lost or inefficiently introduced). This property of IFIT1 could also be advantageous in studies using synthetic RNA (22). This includes applications in which the unmethylated cap is preferred, such as RNA sequencing pipelines using template-switching reverse transcription, in which GpppRNA confers improved efficiency and less bias than m7GpppRNA (22). Therefore, IFIT1 offers complementary properties to those of eIF4E: whereas IFIT1 is not expected to pull-down the cap 1 or cap 2 mRNA of vertebrates (to which our method would thus not apply), it is suitable for enrichment of mRNA lacking methyl groups at the 2′O of the first two nucleotides and facultatively the N7 position in the cap. In such cases, we propose that IFIT1 as a facile and versatile tool could be the preferred alternative to eIF4E for protein-based cap 0-dependent mRNA capture.

We successfully demonstrated on the example of yeast RNA that our IFIT1-based method recovered mRNA in a robust manner while removing the abundant rRNA and non-coding RNA more effectively than the commercial RM method. Unlike eIF4E (10), our IFIT1 method did not require additional ribodepletion, though it is compatible with such downstream purification and could be used in tandem with a ribodepletion step if needed, which would likely further increase sample purity and concentration. Con-B and Cin-S both produced high quality RNA-seq data with an excellent coverage of the protein coding transcriptome. Cin-S and Con-B gave comparable results, with less total read counts but higher mRNA fraction recovered in the Cin-S method, accompanied also by an increased proportion of reads in the region following TSS. This improved selectivity in Cin-S might be due to increased accessibility of IFIT1 in solution, or enabling weak IFIT1 homodimerization which may improve affinity (15,18,19). The main shortcoming of the IFIT1-based approach may be the inability to simultaneously enrich mitochondrial transcripts due to the absence of cap moiety, a downside of any cap-dependent method. Interestingly, even some non-coding RNAs were retained in the IFIT1 pull-down, correlating with their tendency to possess a trimethylated cap structure. IFIT1 may either recognize m_3_^2,2,7^GpppRNA, or, more likely, detect the nascent cap 0 ends of snRNAs and snoRNAs prior to their post-transcriptional trimethylation in the cytosol (32). Although IFIT1 was suggested to be largely sequence-agnostic as seen for its paralog IFIT5 (37) and IFIT1 co-crystal structures with either m^7^GpppAAAA (17) or m^7^GpppAUAGGCGGCG (18), we observed a slight sequence preference of IFIT1, mostly toward adenines. Our work is the first instance of a thorough comparison of IFIT1 sequence specificity on such a large ‘library’ of cap 0 transcripts provided by the yeast transcriptome. Previously, the sequence preference of IFIT1 for RNA was examined quantitatively mostly for the first cap-proximal nucleotide (38), and our results confirm the observed slight preference for adenine over guanosine in the first position (a two-fold difference in the binding constant was previously reported (38)). The sequence preference of IFIT1 mostly coincides with the natural 5′ end-proximal base occurrences in the yeast transcriptome, which are enriched for adenines. This slight bias of IFIT1 was not refractory to obtaining complete sequence coverage and high efficiency in retrieving mRNA counts, since IFIT1 can accommodate any 5′ end sequence in its binding site, and should remain completely agnostic to the sequence of the rest of the mRNA. We conclude that IFIT1 can be used robustly to analyze and compare coding transcriptomes in a largely sequence-independent manner.

The 5′-end capture, ribodepletion and polyA-dependent methods are known to produce slightly different results (4,5,9,30,31) and in this regard our IFIT1 method is expected to differ from RM, and perhaps even more so from the polyA capture as observed in our pilot experiments. We recommend that relative differential analyses should be performed using one methodological approach across all of the compared samples, as is typically done in the case of most RNA-seq analyses. IFIT1 capture should perform uniformly, even regardless of the order of steps, as demonstrated by the high correlation between Cin-S and Con-B data. Importantly, IFIT1-based capture avoided artifacts resulting from the RM procedure, as seen in the depletion of some mRNA (AGA1, MED7 and others), observed also in previous studies (3,39). We attributed these artifacts to off-target hybridization of RM probes directed against rRNA since these mRNAs were not depleted in datasets from other commercial alternatives (2). PolyA-based mRNA capture on oligo-dT is also known to suffer from such off-target probe hybridization artifacts, which may be especially pronounced in the case of AT-rich genomes, and this method may miss numerous polyA(-) transcripts (40). For example, planarian transcriptome studies showed that transposable elements and histone mRNAs were underrepresented in polyA libraries (30). Other known ribodepletion methods may rely on RNase-based rRNA digestion (e.g. by Cas9, RNase H), also directed by hybridizing guide oligonucleotides that may also have putative off-target effects (41–43). The use of RNA-binding proteins such as IFIT1 or eIF4E that may be easily produced in bacteria offers also an economic advantage over any antibody-based approaches. While sequence- or epitope-specific approaches might work well for well-described organisms and conserved motifs, many organisms may require optimization of probes, guides or antibodies. Our IFIT1-based cap 0-dependent capture may therefore offer a more robust, universal approach to mRNA library preparation that may be of value for studies in a wide range of organisms that have cap 0 mRNA. This includes higher plants and lower eukaryotes, for example eukaryotic microorganisms and interesting cases such as slime mold (44). This range may be further broadened by analogous pull-down procedures employing IFIT1 homologs from other species which reportedly also bind cap 1 mRNA (20,21). While the IFIT1-based pull-down can only offer mRNA enrichment (similarly to oligo-dT or eIF4E-based approaches), it is a versatile and a cost-effective capture method that performs on par with the commercially available approaches for analysis of the protein-coding transcriptome.

## DATA AVAILABILITY

Raw data is available under accession number ENA: PRJEB49214 and GEO: GSE210198.

## Supplementary Material

gkac903_Supplemental_Files
