# Supplementary material for: A cap 0-dependent mRNA capture method to analyze the yeast transcriptome
Source: Nucleic Acids Res. 2022 Oct 19;50(22):e132. doi: 10.1093/nar/gkac903 (PMC9825183; doi:10.1093/nar/gkac903)
Supplement: gkac903_Supplemental_Files [file gkac903_supplemental_files.zip › Captions for SuppData_Revision.docx]

**Supplementary Data:**

**Supplementary Data 1.** (**A**) Uniquely mapped reads counted using featureCounts v2.0.3 without normalization. (**B**) Counts normalized to TPM (transcript per million) for all reads. (**C**) Counts normalized to TPM for reads mapped to coding sequences only.

**Supplementary Data 2.** (**A**) Counts normalized with MRN (median ratio normalization) for all reads. (**B**) Counts normalized with MRN for reads mapped to coding sequences only.

**Supplementary Data 3.** Results of Differential Expression analysis performed with the DESeq2 v1.34.0 library. (**A**) Cin-S method compared to RiboMinus. (**B**) Con-B method compared to RiboMinus. (**C**) Con-B method compared to Cin-S. Genes colored in green in the “padj” column are statistically significant (“padj”<=0.01). In the “foldChange” column, among statistically significant genes, those that are decreased at least two-fold (“foldChange”<=0.5) are colored blue and those that are increased at least two-fold (“foldChange”>=2) are colored red.

**Supplementary Data 4.** (**A**) Comparison of Codon Protection Index (from Pelechano, Vicent, Wu Wei, and Lars M. Steinmetz. "Widespread co-translational RNA decay reveals ribosome dynamics." Cell 161(6): 1400-1412. (2015); GEO: GSE63120) for genes relatively depleted in Cin-S (padj <= 0.01; foldChange<=0.5; RiboMinus versus Cin-S): log2 of mean TPM for all Cin-S replicates; log2 of mean TPM for all RiboMinus replicates and foldChange of RiboMinus versus Cin-S. The median of codon protection index for these genes is 0.542 (median of all genes in YPD_5PSeq is 0.536). (**B**) the up-regulated transcripts list from xrn1 mutant (padj <= 0.01; foldChange>=2; WT versus xrn1 mutant) from **Celik, Alper, et al. "High-resolution profiling of NMD targets in yeast reveals translational fidelity as a basis for substrate selection." RNA 23(5): 735-748. (2017); GEO: GSE86428).
