# Supplementary material for: A cap 0-dependent mRNA capture method to analyze the yeast transcriptome
Source: Nucleic Acids Res. 2022 Oct 19;50(22):e132. doi: 10.1093/nar/gkac903 (PMC9825183; doi:10.1093/nar/gkac903)
Supplement: gkac903_Supplemental_Files [file gkac903_supplemental_files.zip › Supplementary Material_Revision2.pdf]

A cap 0-dependent mRNA capture method to analyze the yeast transcriptome

Martyna Nowacka[1,+], Przemysław Latoch[2,3,+], Matylda A. Izert[1], Natalia K. Karolak[1,4], Rafal Tomecki[2,5], Michał Koper[5], Agnieszka Tudek[2], Agata L. Starosta[2*] and Maria W. Górna[1*]

[1] Biological and Chemical Research Centre, Department of Chemistry, University of Warsaw, Warsaw, Warsaw 02-093, Poland
[2] Institute of Biochemistry and Biophysics, Polish Academy of Sciences, Warsaw, Warsaw 02-106, Poland
[3] Polish-Japanese Academy of Information Technology, Warsaw, Warsaw 02-008, Poland
[4] Nencki Institute of Experimental Biology, Polish Academy of Sciences, Warsaw, Warsaw 02-093, Poland
[5] Institute of Genetics and Biotechnology, Faculty of Biology, University of Warsaw, Warsaw, Warsaw 02-106, Poland

[+] contributed equally

[*] To whom correspondence should be addressed. Tel: (+48) 22 55 26 685, Email: mgorna@chem.uw.edu.pl.
Correspondence may also be addressed to (+48) 22 592 33 30, Email: agata.starosta@gmail.com
agata.starosta@ibb.waw.pl

Supplementary Information

Supplementary Box 1. Con-B (“complex on beads”) protocol

Total RNA extraction should be performed according to the available protocols (Phenol-Chloroform, Trizol reagent-based methods or commercial spin column kits).

Con-B protocol

1. Gently shake the bottle in which the Ni Sepharose 6 Fast Flow is supplied until the medium is homogeneous.
2. Transfer 50 µl of slurry from the bottle to Eppendorf tube.
3. Sediment the Ni Sepharose 6 Fast Flow by centrifugation at 500 ×g for 1 min at 4°C.
4. Discard supernatant and replace it with 1 ml of chilled RNase-free water.
5. Gently shake by flipping the tube several times and re-sediment the resins by centrifugation (as before).
6. Discard supernatant and replace with 1 ml of chilled Binding Buffer (50 mM Tris pH 7.5, 150 mM NaCl, 1 mM DTT, 5 mM imidazole, 0.01 % Tween 20, 3 mM $MgCl_2$)
7. Gently shake by flipping the tube several times and re-sediment the resins by centrifugation. Discard supernatant.
8. Add 1 ml of Binding Buffer and 2-3 µg (35-55 pmol) of His-IFIT1 protein to the resin, gently shake (rotate) for 0.5-1h at 4°C.
9. Sediment the resins by centrifugation at 500 ×g for 1 min at 4°C.
10. Discard supernatant and replace it with 1 ml of chilled Binding Buffer.
11. Gently shake by flipping the tube several times and re-sediment the resins by centrifugation. Discard supernatant.
12. Optional: Heat denature RNA samples at 65-70°C for 5-15 min, place on ice for 5 min.
13. Add 1 ml of Binding Buffer, 10 µg of total RNA and poly dIdC (at a final working concentration of 2 µg/ml) to the resins. Gently shake (rotate) for 1h at 4°C.
14. Sediment the resins by centrifugation at 500 ×g for 1 min at 4°C.
15. Discard supernatant and replace it with 1 ml of Wash Buffer (50 mM Tris pH 7.5, 250 mM NaCl, 1 mM DTT, 5 mM imidazole, 0.01 % Tween 20, 3 mM $MgCl_2$).
16. Gently shake by flipping the tube several times and re-sediment the resins by centrifugation.
17. Repeat twice steps 15 and 16. Discard supernatant.
18. Add 400 µl of Wash Buffer and 1 µl (20 µg) of Proteinase K, shake vigorously (800 rpm, shaker) or rotate for 1h at 37°C.
19. Sediment the resins by centrifugation.
20. Transfer supernatant to a new Eppendorf tube and add 2.5 volumes of ethanol. Precipitate overnight at -20°C with the addition of linear acrylamide (at a final working concentration of 10-25 µg/ml). Centrifuge at full speed (13 000 – 16 000 rpm) for 30 min at 4°C. Remove supernatant and wash pellet with 1 ml of ice cold 75 % ethanol. Centrifuge at full speed for 10 min at 4°C. Remove supernatant and dissolve pellet in 10-20 µl of RNase-free H_2O or buffer appropriate for further reactions.
Alternatively, commercial spin column kits can be used for RNA clean-up.
Store samples at -80°C.

Supplementary Box 2. Cin-S (“complex in solution”) protocol

Total RNA extraction should be performed according to the available protocols (Phenol-Chloroform, Trizol reagent-based methods or commercial spin column kits).

Cin-S protocol

1. Heat denature RNA samples at 65-70°C for 5-15 min, place on ice for 5 min.
2. Combine 1 ml of CinS sample containing: Binding Buffer (50 mM Tris pH 7.5, 150 mM NaCl, 1 mM DTT, 5 mM imidazole, 0.01 % Tween 20, 3 mM $MgCl_2$), 2-3 µg (35-55 pmol) of His-IFIT1 protein, 10 µg of total RNA and poly dIdC (at a final working concentration of 2 µg/ml) in an Eppendorf tube. Gently shake (rotate) for 1h at 4°C.
3. In the meantime, gently shake the bottle in which the Ni Sepharose 6 Fast Flow is supplied until the medium is homogeneous.
4. Transfer 50 µl of slurry from the bottle to an Eppendorf tube.
5. Sediment the Ni Sepharose 6 Fast Flow by centrifugation at 500 ×g for 1 min at 4°C.
6. Discard supernatant and replace it with 1 ml of chilled RNase-free water.
7. Gently shake by flipping the tube several times and re-sediment the resins by centrifugation (as before).
8. Discard supernatant and replace with 1 ml of chilled Binding Buffer (50 mM Tris pH 7.5, 150 mM NaCl, 1 mM DTT, 5 mM imidazole, 0.01 % Tween 20, 3 mM $MgCl_2$)
9. Gently shake by flipping the tube several times and re-sediment the resins by centrifugation. Discard supernatant.
10. Add 1 ml of Cin-S sample to the resin, gently shake (rotate) for 0.5-1h at 4°C.
11. Sediment the resins by centrifugation at 500 ×g for 1 min at 4°C.
12. Discard supernatant and replace it with 1 ml of Wash Buffer (50 mM Tris pH 7.5, 250 mM NaCl, 1 mM DTT, 5 mM imidazole, 0.01 % Tween 20, 3 mM $MgCl_2$).
13. Gently shake by flipping the tube several times and re-sediment the resins by centrifugation.
14. Repeat twice steps 12 and 13. Discard supernatant.
15. Add 400 µl of Wash Buffer and 1 µl (20 µg) of Proteinase K, shake vigorously (800 rpm, shaker) or rotate for 1h at 37°C.
16. Sediment the resins by centrifugation.
17. Transfer supernatant to a new Eppendorf tube and add 2.5 volumes of ethanol. Precipitate overnight at -20°C with the addition of linear acrylamide (at a final working concentration of 10-25 µg/ml). Centrifuge at full speed (13 000 – 16 000 rpm) for 30 min at 4°C. Remove supernatant and wash pellet with 1 ml of ice cold 75 % ethanol. Centrifuge at full speed for 10 min at 4°C. Remove supernatant and dissolve pellet in 10-20 µl of RNase-free H_2O or buffer appropriate for further reactions.
Alternatively, commercial spin column kits can be used for RNA clean-up.
Store samples at -80°C.

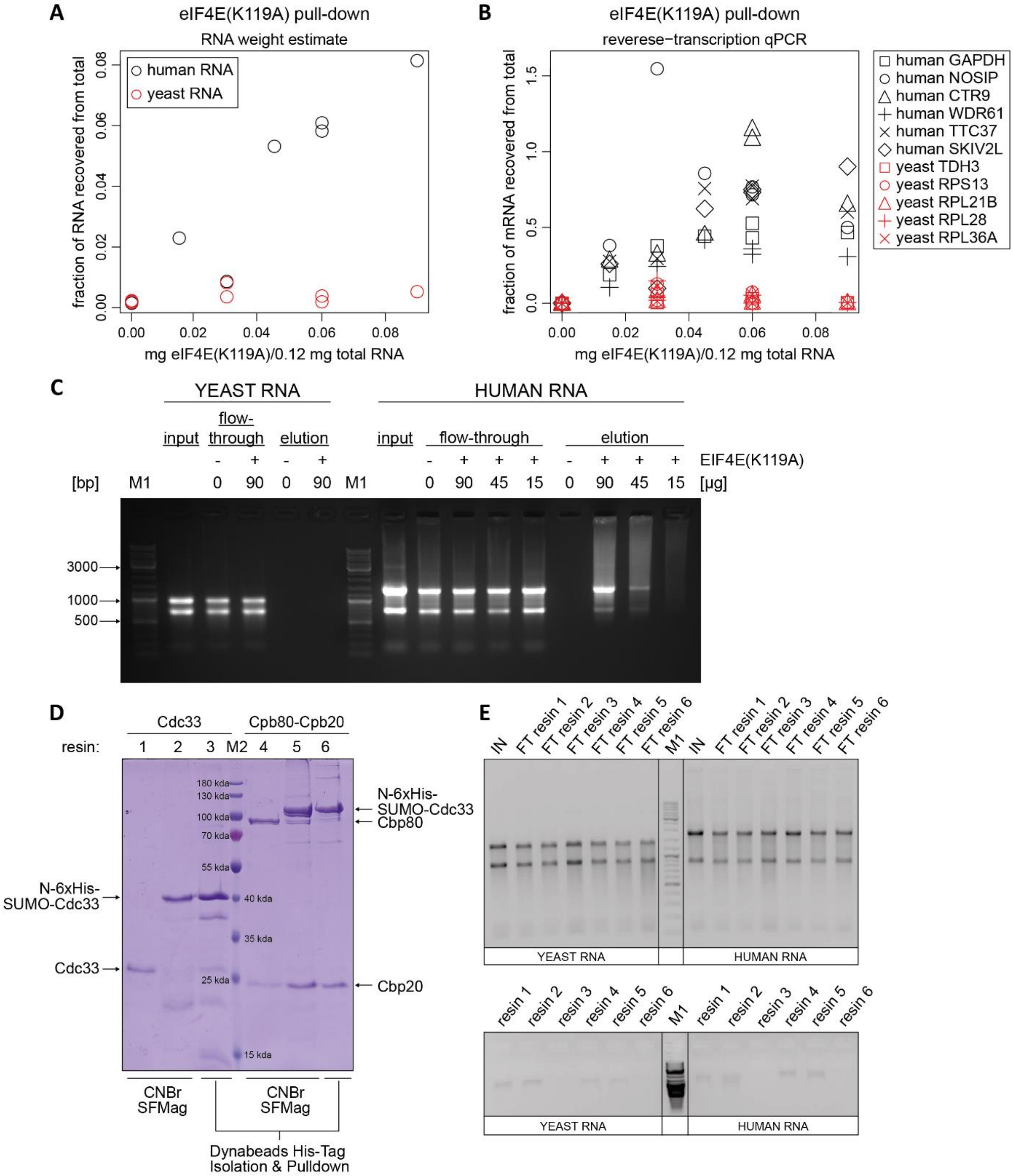

Supplementary Figure S1. RNA capture using eIF4E(K119A). (**A-B**) Changes in the fraction of yeast and human RNA recovered from the total input, as calculated from RNA weight estimates (**A**) or quantified by RT-qPCR (**B**), in relation to the amount of eIF4E(K119A) used as the bait. (**C**) Agarose gel electrophoresis showing the quality and quantity of yeast and human RNAs at various stages of purification using the eIF4E(K119A) bait. Loaded were 2% of the total input and flow-through samples, and 40% of elution samples. M1 - DNA marker (GeneRuler DNA ladder MIX of Life Technology, cat nr SM0331). (**D**) SDS-PAGE analysis (Coomasie staining) of Cdc33 and Cbp80-Cbp20 proteins coupled to the indicated resins. 1 - Cdc33 (no tag), 2 - Cdc33 (N-6xHis-SUMOTag), 3 - Cdc33 (N-6xHis-SUMOTag) , 4 - Cbp80-Cbp20 (no tag), 5 - Cbp80-Cbp20 (N-6xHis-SUMOTag), 6 - Cbp80-Cbp20 (N-6xHis-SUMOTag), M2 - PageRuler Prestained (PageRuler™ Prestained Protein Ladder, 10 to 180 kDa, Life Technology, cat nr 26616). (**E**) Agarose gel electrophoresis showing the result of a binding assay using total yeast RNA and resins 1-6 (upper panel: inputs (IN) and flow-throughs (FT), lower panel eluates).

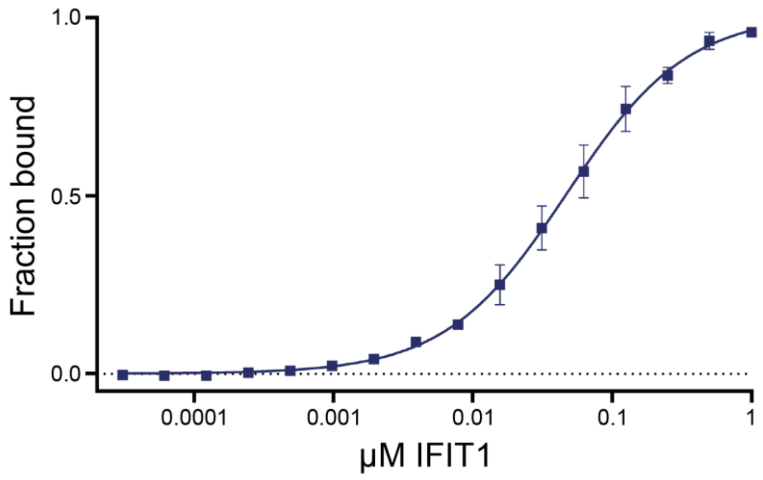

Supplementary Figure S2. Specificity and affinity of IFIT1 for cap 0 RNA. Microscale thermophoresis (MST) measurements suggested apparent K_D = 47 ± 2.4 nM for IFIT1 binding to cap 0 RNA (cap 0-100-mer). Data were analyzed with Graphpad using the one-site specific binding model and the apparent K_D value is reported as the mean ± SEM from a technical triplicate.

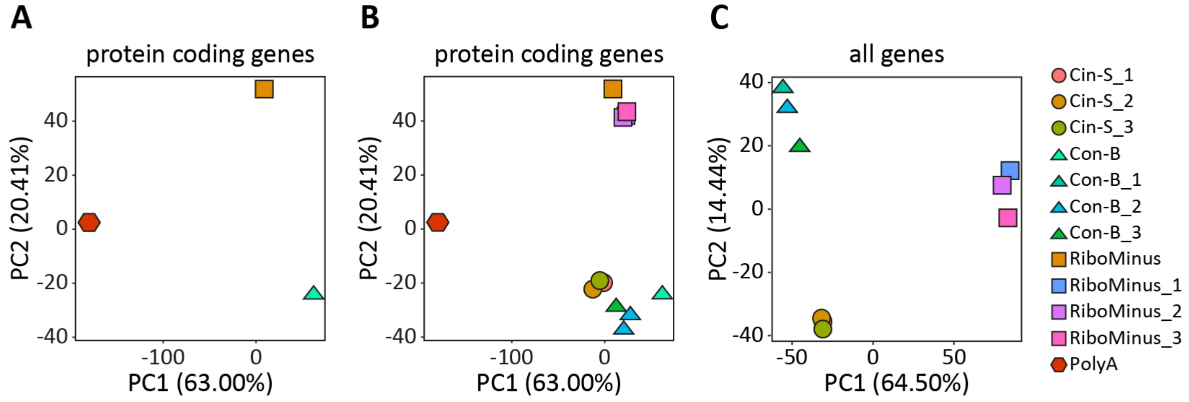

Supplementary Figure S3. PCA of (**A**) protein coding genes included within the first small-scale experiment. (**B**) protein coding genes including the small-scale pre-run and the large-scale NGS analyses; (**C**) all genes from the large-scale experiment. All gene counts were normalized with the median of ratio method.

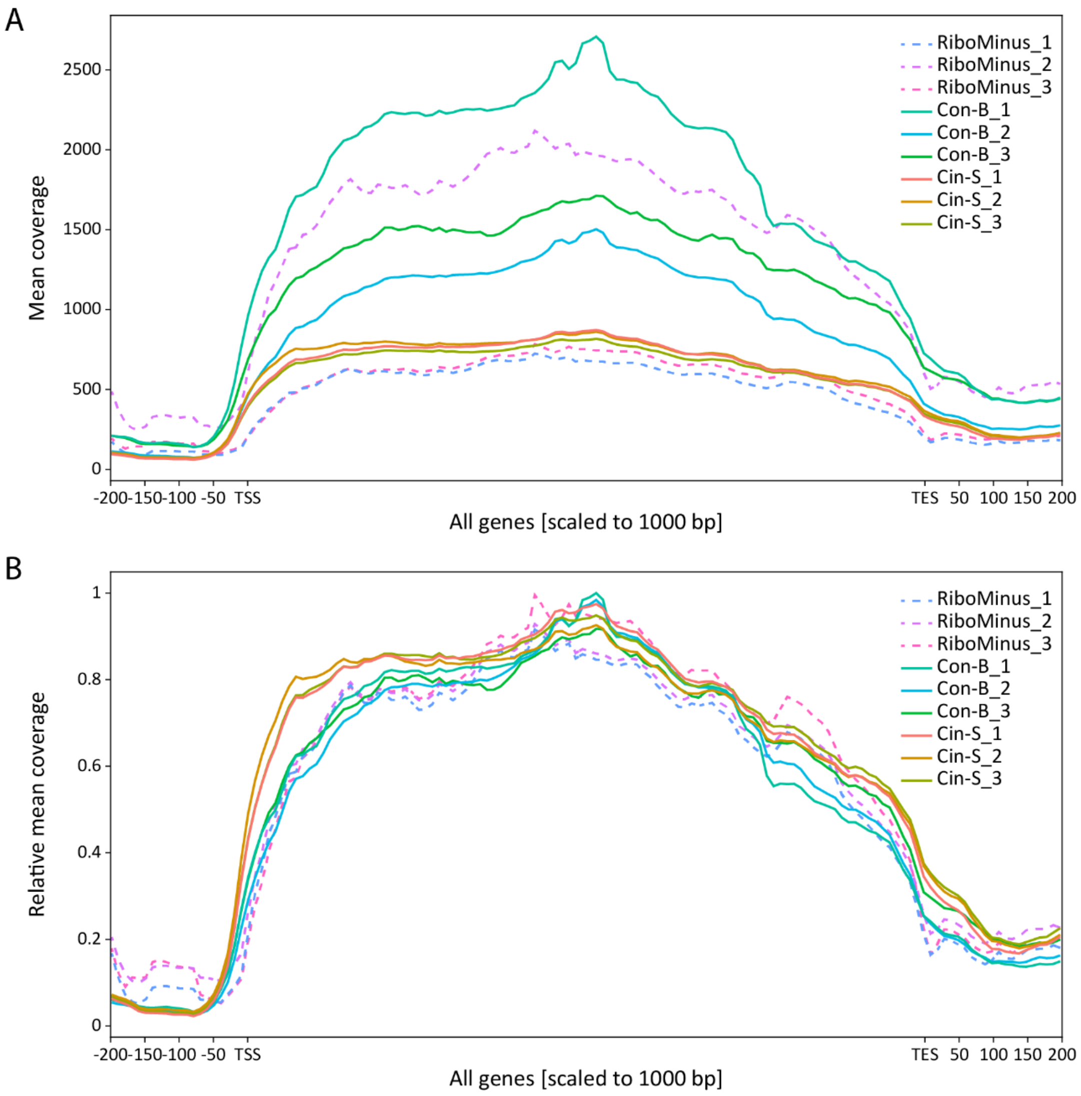

Supplementary Figure S4. (**A**) Mean coverage for all genes with the normalized length of 1000 bp (±200 from Translation Start Sites and Translation End Sites). (**B**) Relative mean coverage for all genes with the normalized length of 1000 bp (±200 from Translation Start Sites and Translation End Sites). Plotted with the deepTools plotProfile function.

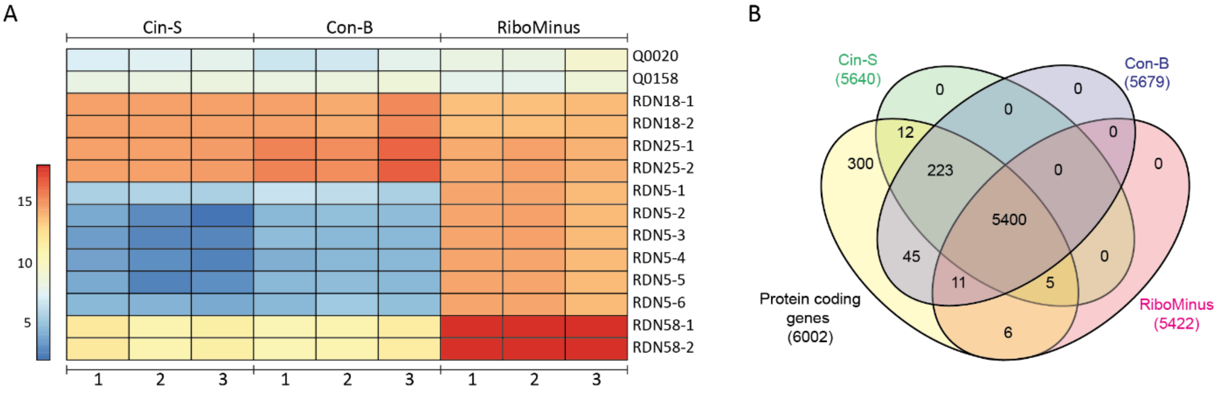

Supplementary Figure S5. (**A**) Heatmap of rRNA genes for all samples and replicates. Scale in log2(TPM+1) including data from the Bowtie2 filtration step. (**B**) Venn diagram of protein-coding genes detected in each method. Only genes with at least 10 raw reads in each replicate were included.

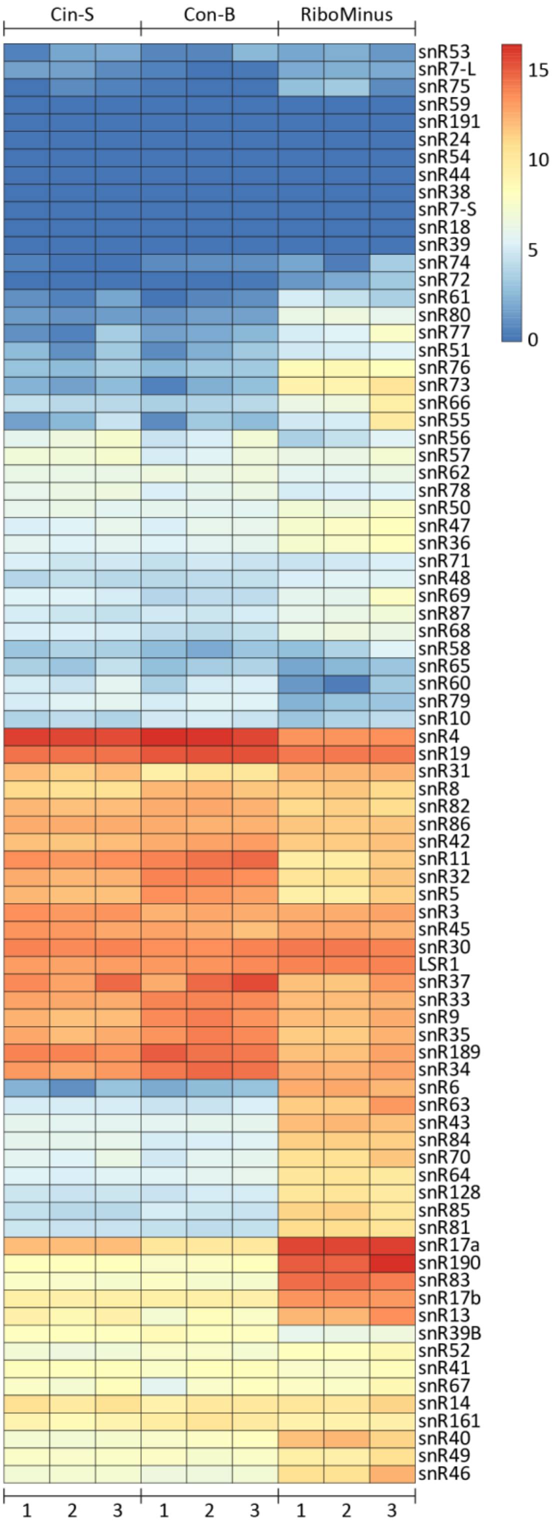

Supplementary Figure S6. Heatmap of snoRNA and snRNA genes for all samples and replicates. Scale in log2(TPM+1).

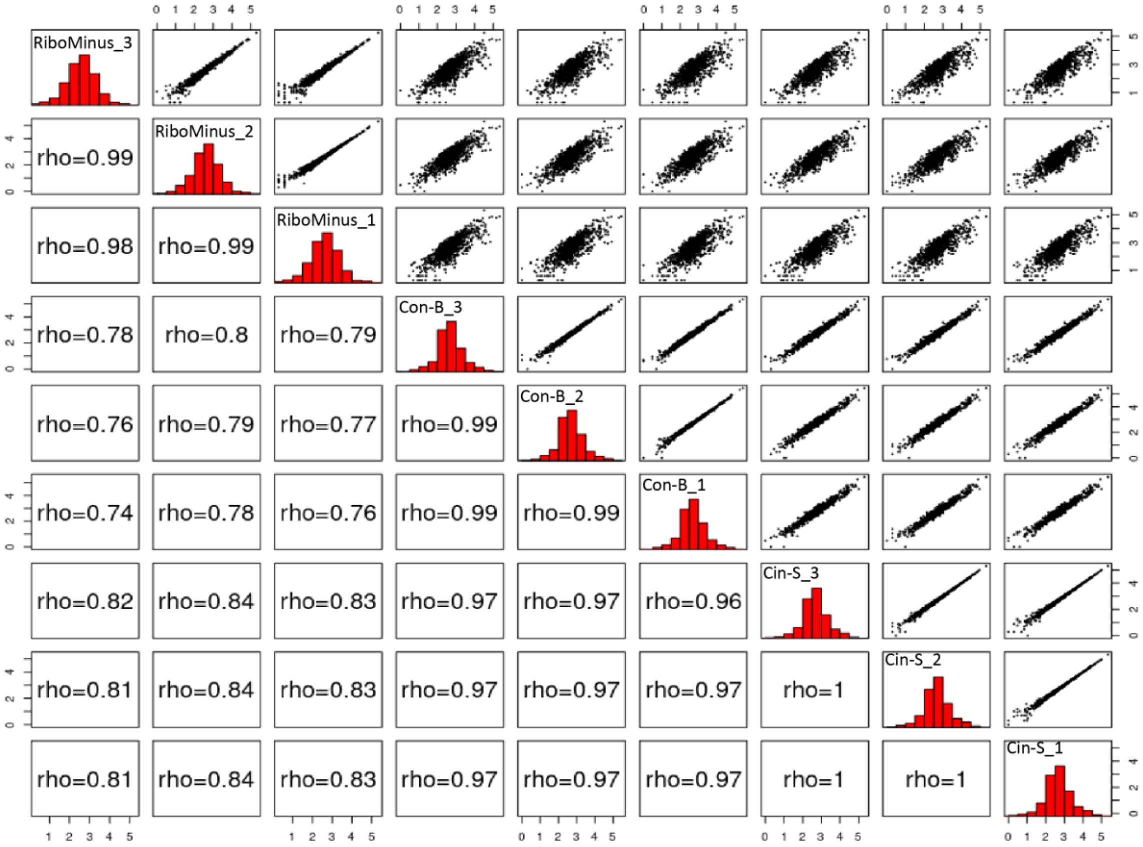

Supplementary Figure S7. Pairwise scatter plots for MRN (median of ratios) normalized protein coding genes counts of all samples and correlation coefficient between them. The upper side of the plot displays log10 normalized scatter plots. The bottom side of the plots is the Pearson correlations of corresponding samples. Diagonal shows the histogram of normalized read counts in the log10 scale.

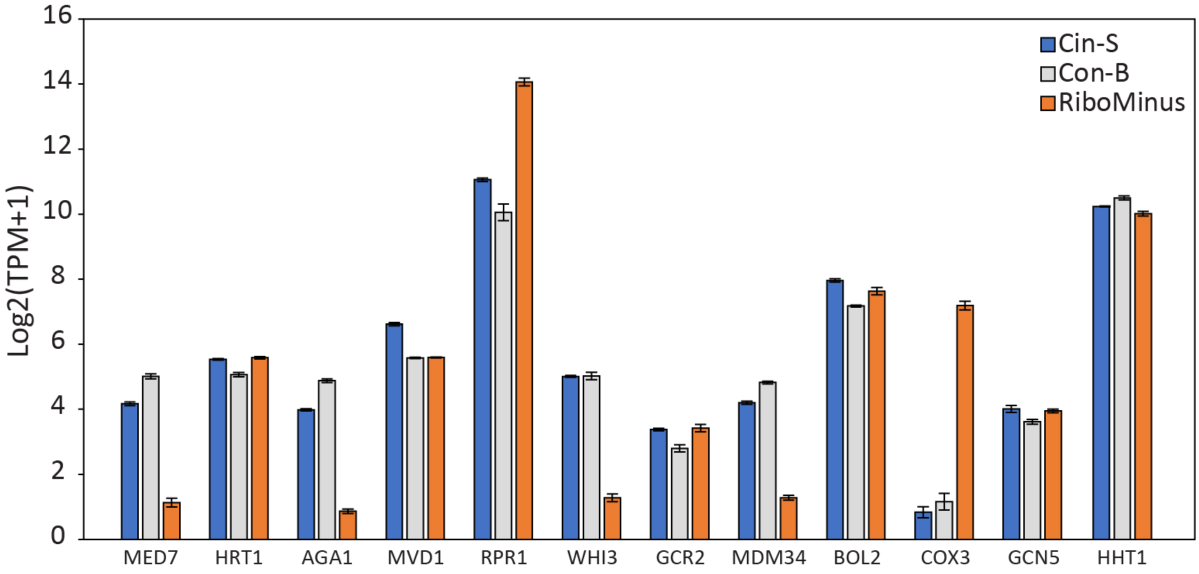

Supplementary Figure S8. The plot of the mean TPM (transcript per million) of all replicates for selected genes in NGS analyses. Scale in log2(TPM+1).

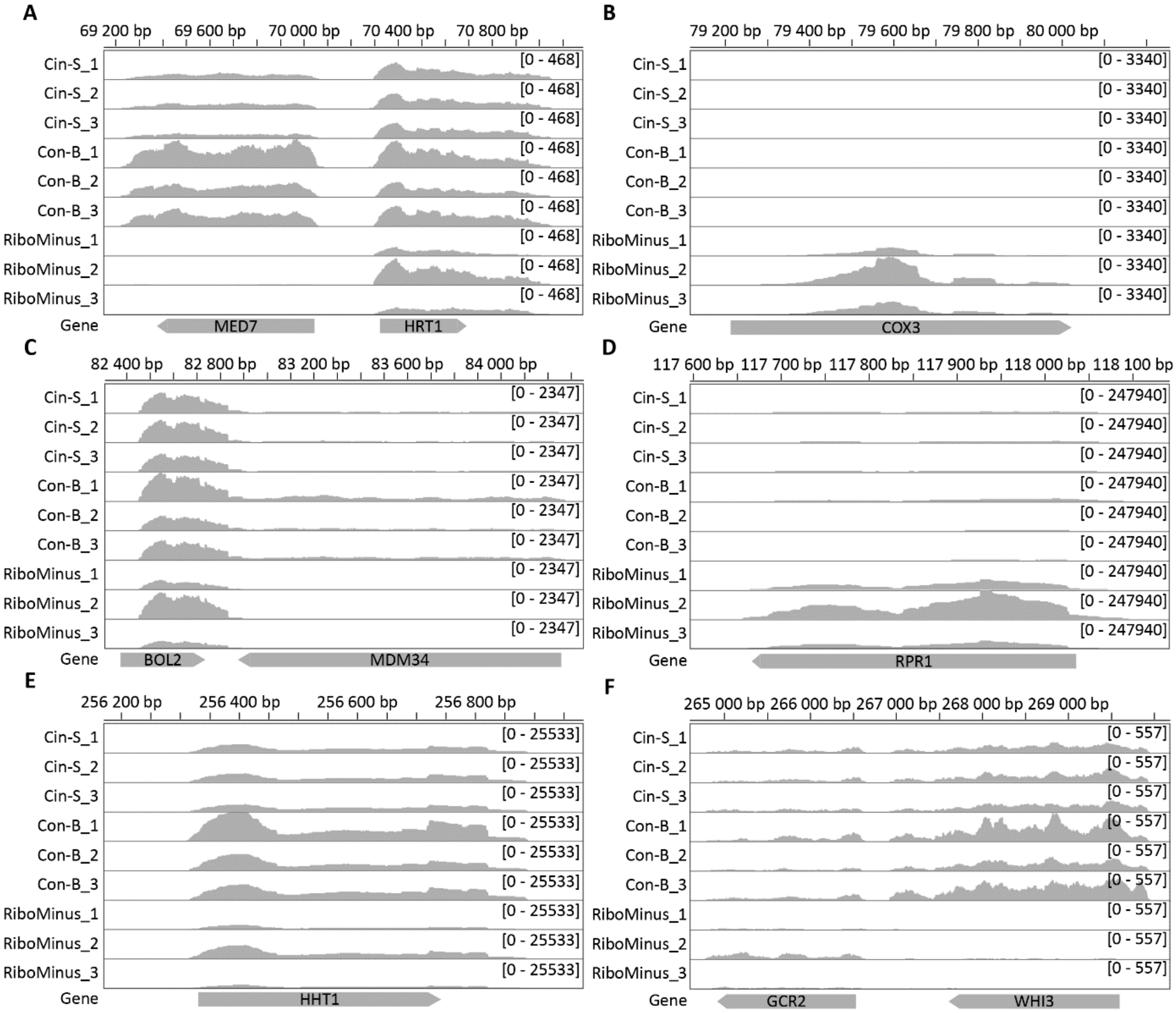

Supplementary Figure S9. Coverage of genes selected for RT-qPCR validation. Each row represents one of the RNA-seq replicates. All panels for specific genes use the same raw counts scale shown on the right side of the plot. (**A**) MED7 and HRT1, (**B**) COX3, (**C**) BOL2 and MDM34, (**D**) RPR1, (**E**) HHT1, (**F**) GCR2 and WHI3.

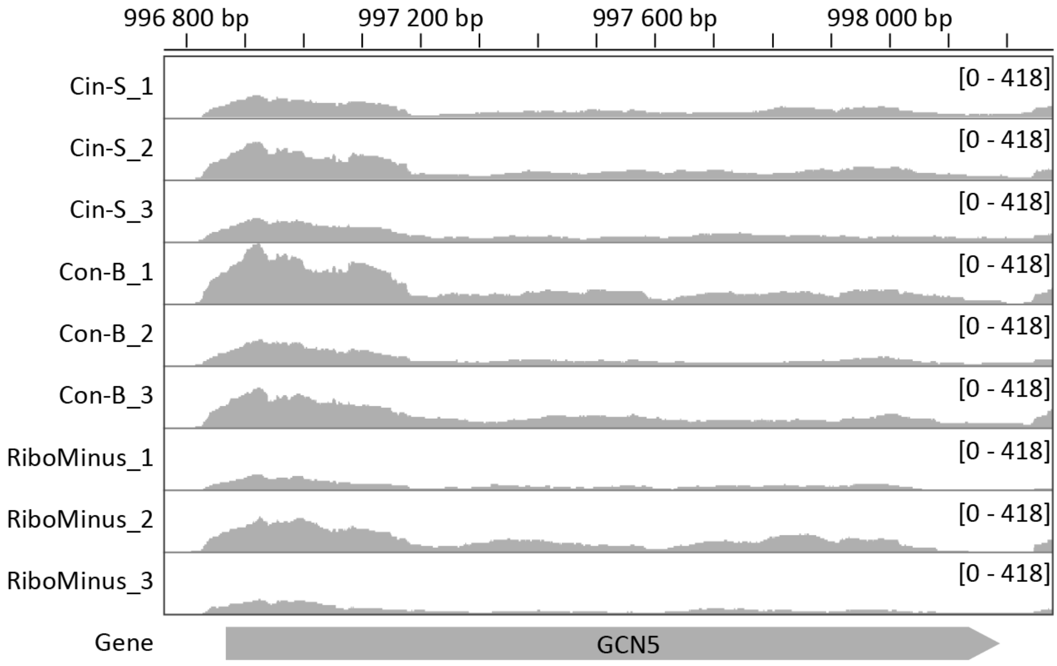

Supplementary Figure S10. Coverage of GCN5 gene. Each row represents one of the RNA-seq replicates. All panels for specific genes use the same raw counts scale shown on the right side of the plot.

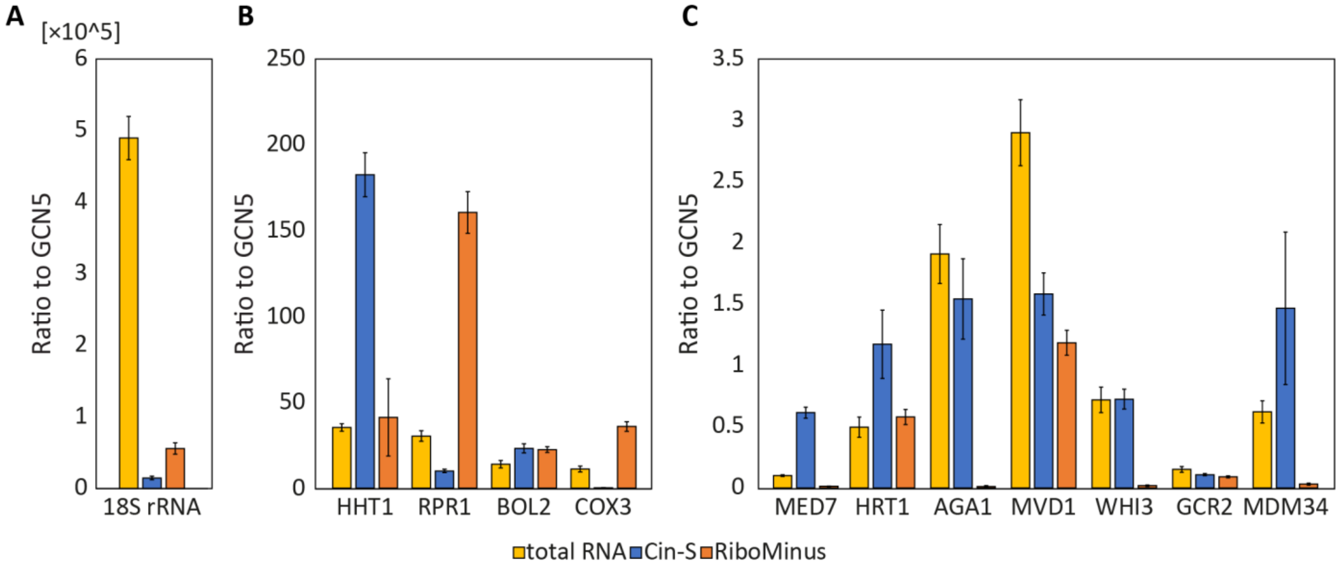

Supplementary Figure S11. RT-qPCR plots of the ratio relative to GCN5 gene for (**A**) 18S rRNA (**B**) HHT1, RPR1, BOL2 and COX3 (**C**) MED7, HRT1, AGA1, MVD1, WHI3, GCR2 and MDM34. The different scales for each graph are shown in the figure.

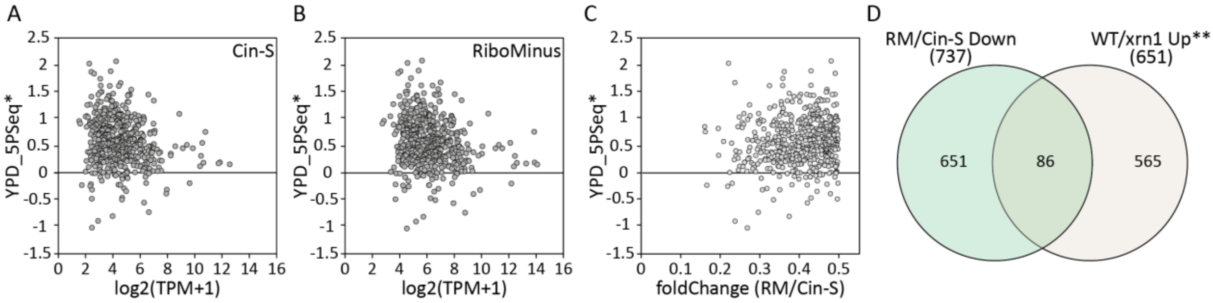

Supplementary Figure S12. Dot plots of codon protection index[1] for genes underrepresented in Cin-S (padj <= 0.01; foldChange<=0.5; RiboMinus versus Cin-S) distributed over: (**A**) log2 of mean TPM for all Cin-S replicates; (**B**) log2 of mean TPM for all RiboMinus replicates; (**C**) foldChange of RiboMinus versus Cin-S.The median of Codon Protection Index for these genes is 0.542 which indicates low co-translational degradation (median of all genes in the YPD_5PSeq set is 0.536). (**D**) A Venn diagram for the comparison of Cin-S underrepresented transcripts (padj <= 0.01; foldChange<=0.5; RiboMinus versus Cin-S) with the up-regulated transcripts from xrn1 mutant[2] (padj <= 0.01; foldChange>=2; WT versus xrn1 mutant).

[1] *Pelechano, Vicent, Wu Wei, and Lars M. Steinmetz. "Widespread co-translational RNA decay reveals ribosome dynamics." Cell 161(6): 1400-1412. (2015); GEO: GSE63120

[2] **Celik, Alper, et al. "High-resolution profiling of NMD targets in yeast reveals translational fidelity as a basis for substrate selection." RNA 23(5): 735-748 (2017); GEO: GSE86428

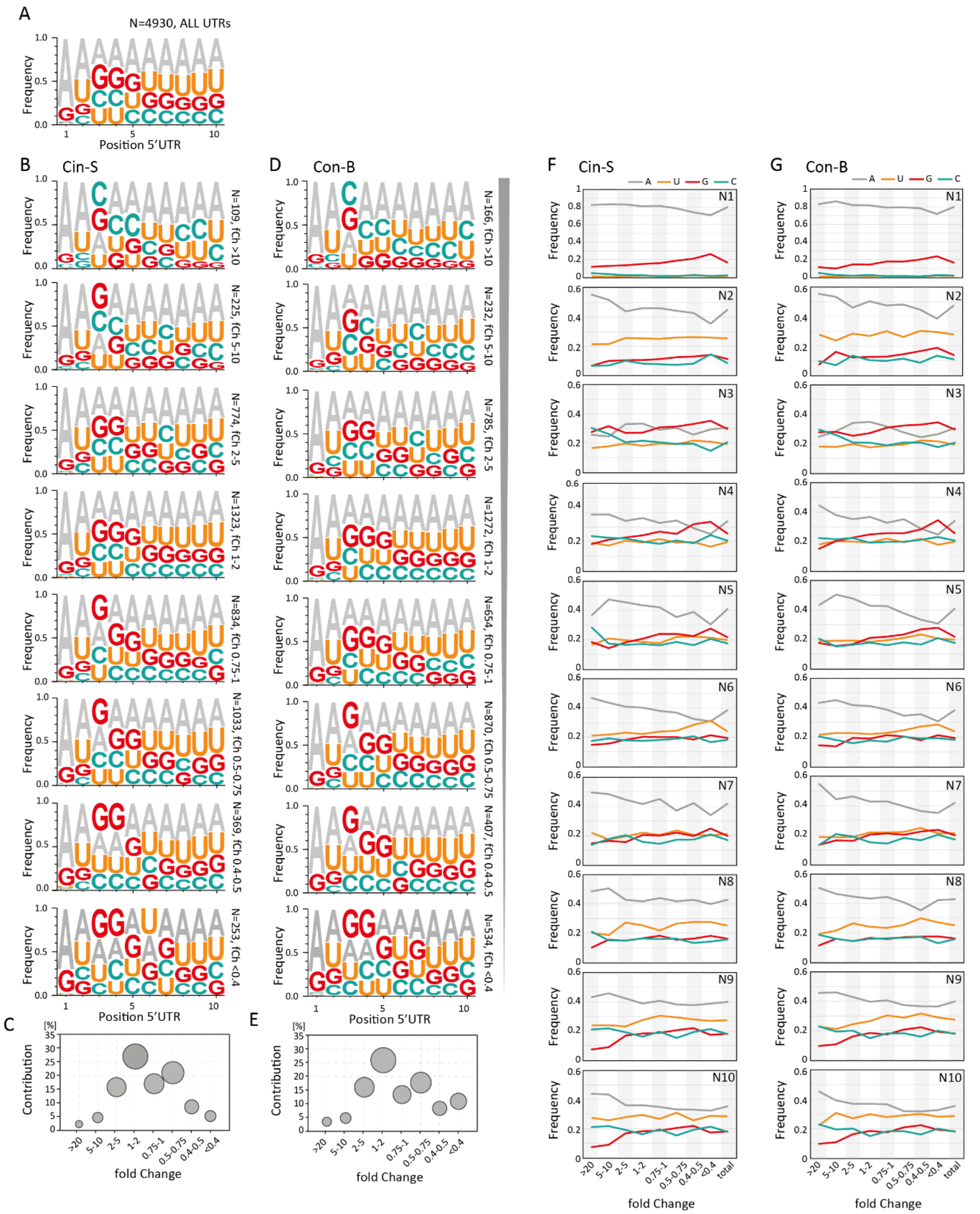

Supplementary Figure S13. Weblogos (v. 3.7.11) of the first 10 nucleotides of the 5'UTRs for (**A**) all 4920 annotated UTRs out of all 5794. Weblogos of ranges determined by the fold change (fCh) values for (**B**) Cin-S data compared to the RM data. (**C**) Contribution of the number of sequences N in the fold Change range group in Cin-S. Weblogos of ranges determined by the fold change (fCh) values for (**D**) Con-B data compared to the RM data. (**E**) Contribution of the number of sequences N in the fold Change range group in Con-B. (**F**-**G**) The frequency of the occurence of each base in the Cin-S (**F**) and Con-B (**G**) datasets for the first 10 nucleotides (N1-N10). The mRNA groups were determined by the fold change (fCh) values compared to RM data. The last data point ("total") represents the average frequency of the bases in all 4920 analysed 5'UTRs.

Supplementary Table 1. Primers used for cloning of yeast protein constructs.

Gene Name_Primer	Oligonucleotide sequence (5'-3')
CDC33_F1	GAAGTCTACCAGGAACAAACCGGTGGATCCATGTCCGTTGAAGAAGTTAGC
CDC33_R1	TCAGTGGTGGTGGTGGTGGTGCTCGAGTTACAAGGTGATTGATGGTTG,
CBP80_F1	TGAAGTCTACCAGGAACAAACCGGTGGATCCATGTTTAATAGAAAAAGAAGAGG
CBP80_R1	ATATATCCTCCTTTAGCGGCCGCTATCTTAAACTTCCTTTGTTTCTTGAATCC
CBP20_F2	TAAGATAGCGGCCGCTAAAGGAGGATATATATGTCCCTGGAAGAATTTGACG
CBP20_R2	GATCTCAGTGGTGGTGGTGGTGGTGCTCGAGTTACTGAGGTACGTAGTTATCATC

Supplementary Table 2. *In vitro* transcribed RNA molecules used for MST and pull-down with IFIT1.

RNA name	Length	5’ moiety	Sequence
80-mer	80 nt	OH	GAAUACACGGAAUUCCUUUUUUUUUUUUUUUUUUUUUUUUAAGAAAGGCAGACUGCCACAUGCAGCGCCUCAUUUGG AUG
100-mer	100 nt	P	GAAUACACGGAAUUCCUUUUUUUUUUUUUUUUUUUUUUUUAAGAAAGGCAGACUGCCACAUGCAGCGCCUCAUUUGG AUGUGUCUGGAGUCUUGGAAGCU
135-mer	135 nt	PPP	GAAUACACGGAAUUCCUUUUUUUUUUUUUUUUUUUUUUUUAAGAAAGGCAGACUGCCACAUGCAGCGCCUCAUUUGG AUGUGUCUGGAGUCUUGGAAGCUUGACUACCCUACGUUCUCCUACAAAUGGACCUUGA
160-mer	160 nt	Cap0	GAAUACACGGAAUUCCUUUUUUUUUUUUUUUUUUUUUUUUAAGAAAGGCAGACUGCCACAUGCAGCGCCUCAUUUGG AUGUGUCUGGAGUCUUGGAAGCUUGACUACCCUACGUUCUCCUACAAAUGGACCUUGAGAGCUUGUUUGGAGGUUCU AGCAGG
Cap 0-100-mer	100 nt	Cap0	GAAUACACGGAAUUCCUUUUUUUUUUUUUUUUUUUUUUUUAAGAAAGGCAGACUGCCACAUGCAGCGCCUCAUUUGG AUGUGUCUGGAGUCUUGGAAGCUUGACUACCCUACGUUCUCCUACAAAUGGACCUUGA

Supplementary Table 3. Statistics of raw sequencing data and adapters trimming.

File	Raw Sequences	Raw Base Pair	Avg. Length of Raw Sequence	Reads with Adapters	Reads with Adapters [%]	Sequences after Trimming	Removed Sequences	Base Pair after Trimming	Trimmed Base Pair [%]	Avg. Length of Sequence after Trimming
**Cin-S_1_R1**	25602045	2585806545	101	9485465	37,05%	25593176	8869	2565104205	0,80%	100,23
**Cin-S_1_R2**	25602045	2585806545	101	9481920	37,04%	25593176	8869	2562763880	0,89%	100,13
**Cin-S_2_R1**	26588949	2685483849	101	9786682	36,81%	26581138	7811	2665065916	0,76%	100,26
**Cin-S_2_R2**	26588949	2685483849	101	9828836	36,97%	26581138	7811	2662704982	0,85%	100,17
**Cin-S_3_R1**	26083259	2634409159	101	9625500	36,90%	26076451	6808	2612607317	0,83%	100,19
**Cin-S_3_R2**	26083259	2634409159	101	9654555	37,01%	26076451	6808	2610602485	0,90%	100,11
**Con-B_1_R1**	66293301	6695623401	101	24873606	37,52%	66216680	76621	6633110212	0,93%	100,17
**Con-B_1_R2**	66293301	6695623401	101	24562291	37,05%	66216680	76621	6617252553	1,17%	99,93
**Con-B_2_R1**	34997311	3534728411	101	13336561	38,11%	34984810	12501	3496231934	1,09%	99,94
**Con-B_2_R2**	34997311	3534728411	101	13268058	37,91%	34984810	12501	3493145977	1,18%	99,85
**Con-B_3_R1**	85616206	8647236806	101	30170234	35,24%	85580057	36149	8575461616	0,83%	100,20
**Con-B_3_R2**	85616206	8647236806	101	30710482	35,87%	85580057	36149	8565352801	0,95%	100,09
**RiboMinus_1_R1**	26929452	2719874652	101	10630914	39,48%	26911574	17878	2653343074	2,45%	98,59
**RiboMinus_1_R2**	26929452	2719874652	101	10685232	39,68%	26911574	17878	2650743417	2,54%	98,50
**RiboMinus_2_R1**	88894589	8978353489	101	35471970	39,90%	88862154	32435	8754277391	2,50%	98,52
**RiboMinus_2_R2**	88894589	8978353489	101	35706514	40,17%	88862154	32435	8748375359	2,56%	98,45
**RiboMinus_3_R1**	25676562	2593332762	101	10496011	40,88%	25644406	32156	2526063550	2,59%	98,50
**RiboMinus_3_R2**	25676562	2593332762	101	10242310	39,89%	25644406	32156	2521667157	2,76%	98,33

Supplementary Table 4. Statistics of rRNA filtering with Bowtie2.

Sample	Sequences after Trimming	rRNA	rRNA [%]	Clean Sequences
**Cin-S_1**	25593176	7527922	29,41%	18065254
**Cin-S_2**	26581138	7482096	28,15%	19099042
**Cin-S_3**	26076451	7846678	30,09%	18229773
**Con-B_1**	66216680	27841262	42,05%	38375418
**Con-B_2**	34984810	13189974	37,70%	21794836
**Con-B_3**	85580057	51849229	60,59%	33730828
**RiboMinus_1**	26911574	17359432	64,51%	9552142
**RiboMinus_2**	88862154	60434208	68,01%	28427946
**RiboMinus_3**	25644406	15145173	59,06%	10499233

Supplementary Table 5. Statistics of mapping to the yeast genome with STAR.

Sample	Total Reads	Uniquely Mapped	Uniquely Mapped [%]	Multimapped	Multimapped [%]	Multimapped Too Many Loci	Multimapped Too Many Loci [%]	Unmapped	Unmapped [%]
**Cin-S_1**	18065254	15972755	88,42%	856295	4,74%	97462	0,54%	1138742	6,30%
**Cin-S_2**	19099042	17108455	89,58%	911906	4,77%	112528	0,59%	966153	5,06%
**Cin-S_3**	18229773	16104539	88,34%	901545	4,95%	121296	0,67%	1102393	6,05%
**Con-B_1**	38375418	33683917	87,77%	2104010	5,48%	521774	1,36%	2065717	5,38%
**Con-B_2**	21794836	19292803	88,52%	1114761	5,11%	263807	1,21%	1123465	5,15%
**Con-B_3**	33730828	26944665	79,88%	2712298	8,04%	557509	1,65%	3516356	10,42%
**RiboMinus_1**	9552142	7761698	81,26%	1161015	12,15%	8698	0,09%	620731	6,50%
**RiboMinus_2**	28427946	22900835	80,56%	3481153	12,25%	33038	0,12%	2012920	7,08%
**RiboMinus_3**	10499233	8354573	79,57%	1165335	11,10%	10915	0,10%	968410	9,22%

Supplementary Table 6. Statistics of reads counting with featureCounts.

Sample	Mapped Reads	Assigned	Assigned [%]	Unassigned Multi Mapping	Unassigned No Features	Unassigned Ambiguity
**Cin-S_1**	18570398	15381593	82,83%	2597643	187865	403297
**Cin-S_2**	19984148	16498054	82,56%	2875693	197180	413221
**Cin-S_3**	19018503	15545929	81,74%	2913964	221173	337437
**Con-B_1**	42018771	31186972	74,22%	8334854	330682	2166263
**Con-B_2**	23596708	18055039	76,52%	4303905	234672	1003092
**Con-B_3**	37144878	25755554	69,34%	10200213	361779	827332
**RiboMinus_1**	10313139	7443370	72,17%	2551441	65640	252688
**RiboMinus_2**	30609007	21958299	71,74%	7708172	200532	742004
**RiboMinus_3**	10951611	8051787	73,52%	2597038	91099	211687

Supplementary Table 7. Summary of biotypes from count data.

Sample	protein_coding	ncRNA	tRNA	snoRNA	telomerase_RNA	snRNA	RNase_P_RNA	SRP_RNA	antisense_RNA	misc_RNA	rRNA	RNase_MRP_RNA
**Cin-S_1**	13449921	1825	2482	1305638	4442	511206	18761	68924	17	311	5529	12537
**Cin-S_2**	14726668	2034	2609	1146455	4560	519247	16716	62457	10	234	6326	10738
**Cin-S_3**	13624503	2002	2224	1298726	4856	511963	17057	66072	5	267	5649	12605
**Con-B_1**	25745953	2630	10100	3559397	3328	1782358	28771	28721	33	601	15491	9589
**Con-B_2**	14475959	1656	5307	2415627	2205	1111858	10451	16425	24	285	7754	7488
**Con-B_3**	20769492	2412	5164	3228830	3394	1669612	10592	36510	43	2541	16062	10902
**RiboMinus_1**	4511011	2263	6737	1008647	1658	362178	84636	1418235	2	692	11090	36221
**RiboMinus_2**	13722342	6284	20941	2911248	4954	1050554	231647	3873027	1	2618	31567	103116
**RiboMinus_3**	4778891	2173	4916	1390525	2519	406428	69834	1329023	0	1538	18411	47529

Supplementary Table 8. Percentage summary of all biotypes for all experimental groups, including the rRNA filtering step.

Group	rRNA	protein_coding	ncRNA	tRNA	snoRNA	telomerase_RNA	snRNA	RNase_P_RNA	SRP_RNA	antisense_RNA	misc_RNA	RNase_MRP_RNA	Multimapped	Unmapped	Unassigned No Features	Unassigned Ambiguity
**Cin-S**	29,23%	53,42%	0,01%	0,01%	4,79%	0,02%	1,97%	0,07%	0,25%	0,00%	0,00%	0,05%	3,84%	4,10%	0,77%	1,47%
**Con-B**	49,75%	32,65%	0,00%	0,01%	4,93%	0,00%	2,44%	0,03%	0,04%	0,00%	0,00%	0,01%	3,89%	3,59%	0,50%	2,14%
**Ribo Minus**	65,76%	16,27%	0,01%	0,02%	3,76%	0,01%	1,29%	0,27%	4,68%	0,00%	0,00%	0,13%	4,14%	2,55%	0,25%	0,85%

Supplementary Table 9. Primers used for RT-qPCR analysis

Gene Name_Primer Direction	Oligonucleotide sequence (5'-3')	Primer Efficiency	Amplicon length (nt)	Gene accession number
Yeast				
MED7_F	ACCAACTGCCCGATTTAGAG	2.05	147	AY692889.1
MED7_R	CGCCGATGAGTTCCAAGTAA	2.05		
HRT1_F	GCTGTTGACAACTGTGCTATTT	2.06	105	NM_001183387
HRT1_R	CCAGGCTGCTACACATTCA	2.06		
AGA1_F	CACCACCTTATCTGTGACTTCC	1.97	101	Z71659.1
AGA1_R	CCACGGTTGTAGTTCCTACTTC	1.97		
GCN5_F	ATGAAGGTGGTACGCTGATG	1.98	92	Y693215.1
GCN5_R	CAGGGCCGCTTCTTGTAATA	1.98		
MVD1_F	TAGATCGTTGTTTGGCGGATAC	1.99	94	AY693152.1
MVD1_R	GTCAGAGCTGTCTGCGATTT	1.99		
WHI3_F	CACAACAGCCACAACAACAC	2.06	101	NM_001183035
WHI3_R	AACCTGGCCTTGAGAAGATAAC	2.06		
GCR2_F	AAACGATGAATTGTGGCGATTAG	2.05	91	NM_001183037
GCR2_R	CTTCCTGCGTCCTTGAGTTAG	2.05		
HHT1_F	CAGGTACTGTTGCTTTGAGAGA	2.02	144	AY558343.1
HHT1_R	CACCGATGGCAGAAGATTGA	2.02		
MDM34_F	AACAGGATACACCCAGCTTTAC	1.96	135	NM_001181084
MDM34_R	TCCCAATTCCAGGGTTCTTTAC	1.96		
BOL2_F	CGAGAGGATAGAATCAGCCATAC	1.92	99	AY558390.1

BOL2_R	TCGCTGACCACCACAATATC	1.92		
RPR1_F	CTTGTCCTCCGGGTTAATGT	1.99	97	NR_132166.1
RPR1_R	TCTGATAACAACGGTCGGTAAA	1.99		
COX3_F	AGCTGTACAACCTACCGAATTAC	2.04	97	KP263414.1
COX3_R	ACCTGCGATTAAGGCATGAT	2.04		
18SrRNA_F	CCTTTACTACATGGTATAACTGTGG	1.82	119	NR_132222
18SrRNA_R	AATCATCAAAGAGTCCGAAGACATTG	1.82		
TDH3_F	CTCTCACTCTTCCATCTTCGAT	2.04	77	AY557831.1
TDH3_R	CGTACCAGGAGACCAACTT	2.04		
RPS13_F	ATTTCTTCTTCTGCTATTCCATACTCT	2.08	101	YDR064W
RPS13_R	CCCTTTCTCGCGTACTTGAC	2.08		
Rpl21B_F	ACAGATCTCGTACACGTTACA	1.79	102	YPL079W
Rpl21B_R	CGACAATGTCACCAACCT	1.79		
RPL28_F	CGGTAAAGGTCGTATCGGT	2.03	87	YGL103W
RPL28_R	TCCATGTTAATTCTGTGGTGATGT	2.03		
Rpl36A_F	AAGGTAAGAAGGTCACTAGCA	2.20	68	YMR194W
Rpl36A_R	GTTGGAAGCAGCACCTTT	2.20		
Human				
GAPDH_F	TTGCCATCAATGACCCCTTCA	2.35	76	CCDS8549.1
GAPDH_R	AATTTGCCATGGGTGGAATCA	2.35		
WDR61_F	GCAAGCCCATGATGATGCCATTTGG	2.13	306	CCDS10300.1
WDR61_R	AAGGCCAAAGTCCAGGCATCCAC	2.13		

CTR9_F	AGATTTGATTTGGCCCTTGCTG	1.84	99	CCDS7805.1
CTR9_R	TTCATCTTGTTTGCGTGCCC	1.84		
TTC37_F	CTGTCTGCACAAGTGGATGG	1.97	127	CCDS4072.1
TTC37_R	CTGTTACCAGACGCACCAAG	1.97		
NOSIP_F	TGGTGGACCCTGTGACTGGAGAC	1.98	115	CCDS12772.1
NOSIP_R	GGCCGTGATTTCTCCGCTTGC	1.98		
SKIV2L_F	GATTGCTGCCTTGCTCTC	2.25	129	CCDS4731.1
SKIV2L_R	ACCTGGACCTCACCAATC	2.25		
